# Genet-specific DNA methylation probabilities detected in a spatial epigenetic analysis of a clonal plant population

**DOI:** 10.1371/journal.pone.0178145

**Published:** 2017-05-22

**Authors:** Kiwako S. Araki, Takuya Kubo, Hiroshi Kudoh

**Affiliations:** 1 Center for Ecological Research, Kyoto University, Otsu, Shiga, Japan; 2 Department of Biotechnology, Faculty of Life Sciences, Ritsumeikan University, Kusatsu, Shiga, Japan; 3 Faculty of Environmental Earth Science, Hokkaido University, Sapporo, Japan; Chinese Academy of Sciences, CHINA

## Abstract

In sessile organisms such as plants, spatial genetic structures of populations show long-lasting patterns. These structures have been analyzed across diverse taxa to understand the processes that determine the genetic makeup of organismal populations. For many sessile organisms that mainly propagate via clonal spread, epigenetic status can vary between clonal individuals in the absence of genetic changes. However, fewer previous studies have explored the epigenetic properties in comparison to the genetic properties of natural plant populations. Here, we report the simultaneous evaluation of the spatial structure of genetic and epigenetic variation in a natural population of the clonal plant *Cardamine leucantha*. We applied a hierarchical Bayesian model to evaluate the effects of membership of a genet (a group of individuals clonally derived from a single seed) and vegetation cover on the epigenetic variation between ramets (clonal plants that are physiologically independent individuals). We sampled 332 ramets in a 20 m × 20 m study plot that contained 137 genets (identified using eight SSR markers). We detected epigenetic variation in DNA methylation at 24 methylation-sensitive amplified fragment length polymorphism (MS-AFLP) loci. There were significant genet effects at all 24 MS-AFLP loci in the distribution of subepiloci. Vegetation cover had no statistically significant effect on variation in the majority of MS-AFLP loci. The spatial aggregation of epigenetic variation is therefore largely explained by the aggregation of ramets that belong to the same genets. By applying hierarchical Bayesian analyses, we successfully identified a number of genet-specific changes in epigenetic status within a natural plant population in a complex context, where genotypes and environmental factors are unevenly distributed. This finding suggests that it requires further studies on the spatial epigenetic structure of natural populations of diverse organisms, particularly for sessile clonal species.

## Introduction

Clonal organisms repeatedly produce genetically identical individuals, and this type of asexual propagation is common across plants, animals, fungi, and bacteria [1, 2]. Clonality results in hierarchies of individuality, and genetic individuals do not necessarily correspond to physiological ones. In clonal plants, an individual or a group of individuals that originate from a single zygote is referred to as a *genet*. A single genet often consists of multiple *ramets* (morphologically determined units of individuals that can exist as physiologically independent individuals if separated), and often spreads across heterogeneous environments [1, 3, 4]. Because plants are sessile organisms, the spatial distribution of genets within populations, often referred to as the clonal/genetic population structure, displays long-lasting patterns that have significant consequences for the adaptation of clonal plants [5–7]. Many studies have analyzed the spatial genetic structure of clonal plant populations and identified diverse patterns of genet distribution [8–11]. In some species, a single genet can cover tens or hundreds of square meters as a result of the recruitment of genetically identical offspring across multiple clonal generations [8, 12, 13].

Epigenetic population structure is an emerging topic in the literature on the genetic makeup of plant populations [14–20]. Although the spatial genetic structures of populations have been analyzed in diverse plant species since the first reports on a clonal plant population in the 1970s (e.g. [21]), studies on the spatial epigenetic structure of plant populations have only been carried out more recently. For example, Richard et al. [20] found large epigenetic differentiation among genetically less-differentiated populations of an invasive *Fallopia* species. Because the accumulated information on the spatial genetic structures of populations has been critical for advancing our understanding of plant population biology, we can now expect further significant advances if similar efforts are made to analyze the spatial epigenetic variation that should exist at both the ramet and genet levels.

Epigenetic variation is based on several processes, such as DNA methylation and histone modification. These processes often result in mitotically or meiotically heritable changes in gene function without altering DNA sequences [22–26]. Methylation-sensitive amplified fragment length polymorphism (MS-AFLP) is a useful method for detecting DNA cytosine methylation, one of the major epigenetic regulations for which the detailed mechanisms have recently been revealed [27, 28]. The MS-AFLP method has been applied in the model plant *Arabidopsis thaliana* [29] and in crops such as maize [30], tobacco [31], rice [32, 33], and cotton [34, 35] as well as in natural plant populations [14–17, 20].

Although epigenetic variation does not require DNA sequence polymorphisms, some epigenetic states are under the rigorous control of the genotypic background [36, 37]. In *A*. *thaliana* and other plants, genotypic control of some MS-AFLP variation has been detected under experimental conditions [29, 37]. On the other hand, previous reports have shown that environmental stimuli alter epigenetic states [38–41]. Furthermore, responses of epigenetic status to environmental factors may persist long after the initiating factor has disappeared [18, 24, 42]. In other examples, probabilistic changes in epigenetic states and transmission create a patchy distribution of distinct epigenetic states among cells [22, 43]. The labile yet heritable nature of epigenetic variation, however, leads us to assume that its control is partly deterministic and partly stochastic. Yet we do not know whether these genotypic and environmental effects on epigenetic status are significant under natural conditions.

In clonal plant populations, individuals with shared genotypes (i.e. genets) are distributed across spatially heterogeneous environments. By performing epigenetic analyses on a clonal plant population, it is possible to evaluate whether or not epigenetic variation corresponds to the spatial distribution of genets and/or environmental factors in natural habitats. In addition, we need to develop a statistical framework that can appropriately handle the probabilistic nature of epigenetic variation. The hierarchical Bayesian method allows us to estimate epigenetic status in terms of probabilistic distributions, rather than as scalar values, and simultaneously to evaluate the dependency of epigenetic variation on genets and environmental factors [44].

Here, we report an investigation of the spatial epigenetic population structure of a clonal plant, *Cardamine leucantha* (Brassicaceae). Using combined microsatellite (SSR) polymorphism and MS-AFLP analyses of more than 300 ramet samples with known spatial locations, we determined the distribution of genets as well as their methylation probabilities in a clonal plant population. We addressed the following questions: (1) How is epigenetic variation distributed spatially in a clonal plant population? (2) How much of the spatial pattern of epigenetic variation is explained by genet distribution and by the spatial heterogeneity of environments?

### Conceptual framework

**Fig 1:**
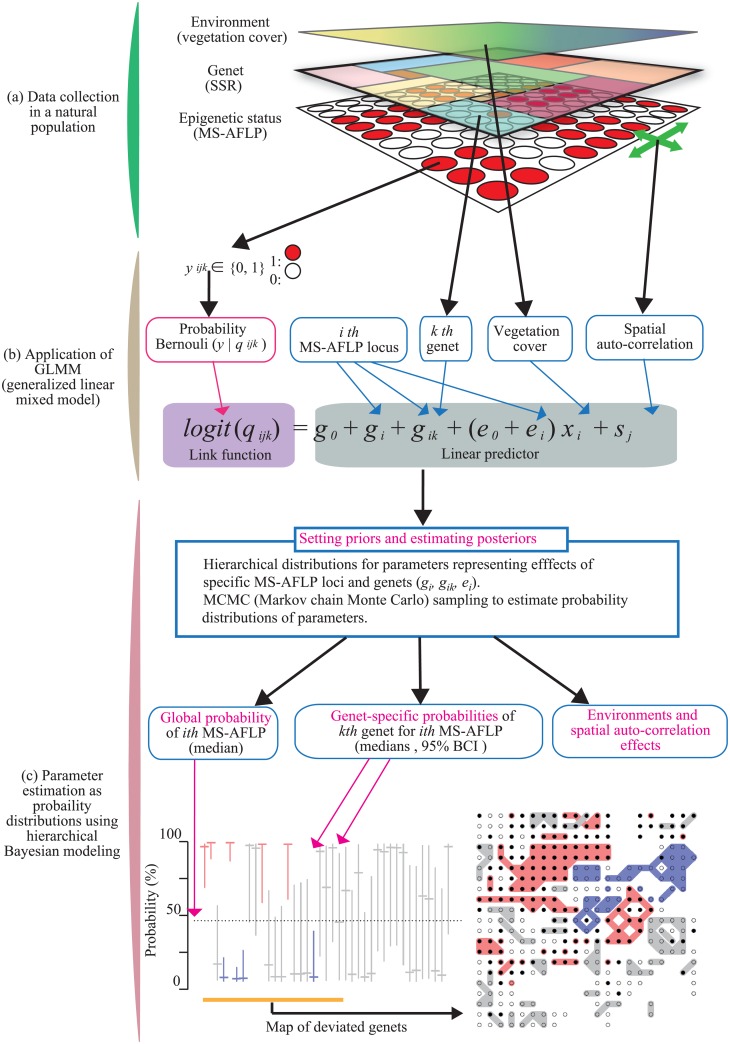
Conceptual framework for analyses of spatial epigenetic structure in a clonal plant population. The analyses consisted of three conceptual parts (a, b, and c). (a) From the natural population, we obtained three types of spatial information: environmental heterogeneity (vegetation cover), genet distribution (SSR), and epigenetic variation (MS-AFLP). In the top layer, spatial environmental heterogeneity is represented by color gradients. In the second layer, different genets are indicated by different colors. In the bottom layer, the epigenetic status of a particular epigenetic site in each sampled ramet is indicated by either closed (positive subepiloci) or open (zero subepiloci) circles. (b) GLMM (generalized linear mixed model), the epigenetic status *y*_*ijk*_ ∈{0, 1} was assumed to follow the Bernoulli distribution of probability, *q*_*ijk*_. This is defined using a combination of a logit link function and a linear predictor, *logit*(*q*_*ijk*_) = *g*_0_ + *g*_*i*_ + *g*_*ik*_ + (*e*_0_ + *e*_*i*_) *x*_*j*_ + *s*_*j*_ (see text for details). (c) To represent the probabilistic nature of epigenetics, we estimated model parameters as probability distributions using hierarchical Bayesian modeling and MCMC (Markov chain Monte Carlo) computation. We defined and compared two kinds of probabilities: the global probability of the *i*th MS-AFLP loci, and the genet-specific probability of *k*th genet for the *i*th MS-AFLP loci. The results are visualized using figures for each MS-AFLP locus as shown in the bottom diagrams. In the bottom left graph, methylation probability (*q*) is converted from *logit* (*q*) [*logit* (*q*) = *log* (*q* /(1 –*q*)), *q* = 0.0067, 0.27, 0.5, 0.73, and 0.99 corresponds to *logit* (*q*) = –5, –1, 0, 1, and 5, respectively]. In the bottom left graph, medians of the global probability (%) are shown by a horizontal dotted line. Medians and 95% BCI of genet-specific probabilities are shown by horizontal and vertical lines, respectively. Genets are arranged from left to right in decreasing order of number of ramets. The spatial locations of the genets are shown in the bottom right panel. For both of the lower panels, red and blue coloring represents genets that deviate positively and negatively from the global probability, respectively.

In this study, the primary focus of the integrated spatial analysis of environment, genet, and epigenetic status (vegetation cover, SSR, and MS-AFLP, respectively) was to identify ‘genet effects’ (effects of genet distribution) on spatial epigenetic variation in a natural clonal plant population (Fig 1a). It should be noted that genet effects involve both genotype effects and environmental/stochastic effects shared by the ramets within genets. The latter may include long-term environmental effects that clone members experience at an earlier stage of clonal spread. Genetic and environmental variation are two potential major determinants of epigenetic variation. Common garden experiments are often designed to separate these determinants statistically, because analyses in a natural population are not straightforward. In natural populations, neither genets nor environments are randomly distributed, and independence of the epigenetic states of neighboring ramets cannot be assumed either.

To represent the complex nature of a natural population, we applied a generalized linear mixed model (GLMM) to epigenetic variation (Fig 1b). We treated epigenetic status as a probability. The response variable MS-AFLP at locus *i* in sample *j* in genet *k* is defined as *y*_*ijk*_ ∈ {0,1}, in which the values 0 and 1 represent any binary variables corresponding to the methylation states; in this article, we applied the mixed scoring procedure [45] (see Materials and methods for details). The epigenetic status *y*_*ijk*_ is assumed to follow the Bernoulli distribution of probability *q*_*ijk*_. To relate *q*_*ijk*_ to a linear predictor, we used a logit link function, *log* (*q*_*ijk*_/(1 − *q*_*ijk*_)), hereafter *logit* (*q*_*ijk*_) (Fig 1b). *Logit* (*q*) is the natural logarithm of the odds ratio of being 0 vs. 1 methylation states; it becomes zero when *q* = 0.5, and takes positive and negative infinite values when *q* approaches 0 and 1, respectively. We modeled *logit* (*q*_*ijk*_) as follows:

$$logit\;\left(q_{ijk}\right)=g_{0}+g_{i}+g_{ik}+\left(e_{0}+e_{i}\right)x_{j}+s_{j},$$

where *g*_0_ is the intercept of the linear predictor, and *g*_*i*_ and *g*_*ik*_ are deviations in MS-AFLP at locus *i* across all genets (global) and in genet *k* (genet-specific), respectively (Fig 1b). The explanatory variable *x*_*j*_ represents local vegetation cover and has the coefficients *e*_0_ and *e*_*i*_, which are the common slope for all loci and the deviation in locus *i*, respectively. The term *s*_*j*_ represents the random effects of sampling point *j*, including spatial random effects (Fig 1b; see Materials and methods for details).

We used hierarchical Bayesian modeling to estimate parameters in the GLMM as probability distributions rather than as scalar values (Fig 1c). For a particular set of binary scores of MS-AFLP polymorphic loci, we estimated both global probability and genet-specific probability. Global probability is the overall probability that a specific MS-AFLP score is one, and genet-specific probability is estimated for each genet with multiple ramets (Fig 1c). The hierarchical Bayesian approach allows us to estimate genet-specific probabilities for a specific methylation state of multiple genets simultaneously [44]. There are two types of distribution for parameters representing the effects of specific MS-AFLP loci and genets: prior and posterior distributions. Posterior distributions are estimated based on the combinations of likelihood function, data, and prior distributions. For each type of scores of MS-AFLP loci, we estimated the posterior distributions of global and genet-specific probabilities using the Markov chain Monte Carlo (MCMC) method (Fig 1c; see Materials and methods for details). When the 95% Bayesian confidence intervals (BCI) of genet-specific probabilities did not include the global probability (median), we treated it as an indication of genet-specific effects on epigenetic status. These analyses allowed us to detect and map genet-specific effects on epigenetic states.

Environmental variation can affect epigenetic states as well as indirect genet effects, and the model allows us to detect such an effect, if it exists (represented by the term (*e*_0_ + *e*_*i*_)*x*_*i*_; Fig 1b and 1c). In addition, the epigenetic states of neighboring ramets may not be independent, as a result of unknown factors not specifically modeled. Our model incorporated positive spatial autocorrelation between neighboring ramets (*s*_*i*_, Fig 1b and 1c). Therefore, our analyses do not necessarily assume spatial independence in epigenetic variation.

## Materials and methods

### Study species and site

**Fig 2:**
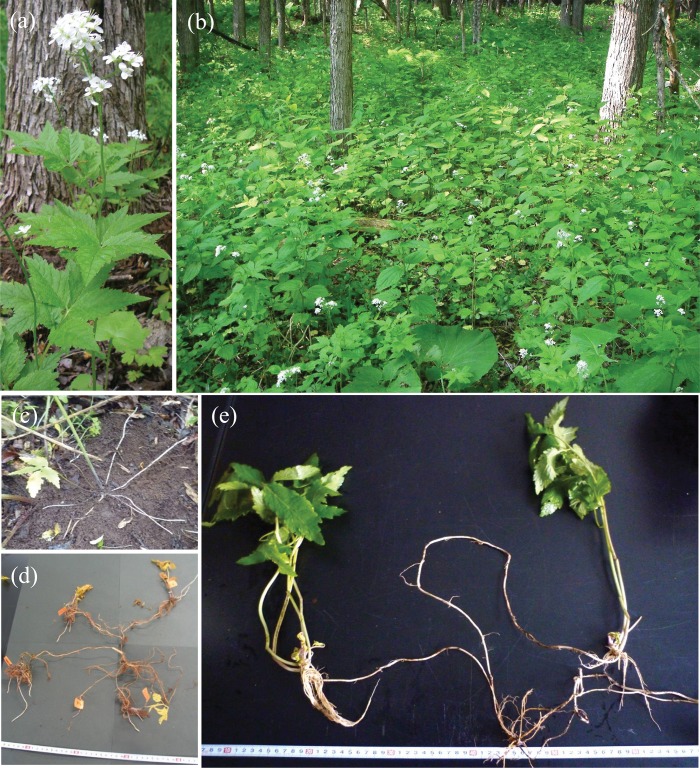
A flowering ramet of *Cardamine leucantha* (a), the study population at Rikubetsu, Hokkaido, Japan (b), and typical clonal growth of the study species (c–e). Multiple underground stolons (stoloniferous rhizomes) begin to elongate in spring (c). Daughter ramets (tagged with orange tape) are formed at the tip of stolons in late autumn (d). Multiple daughter ramets produced from a single mother ramet extend shoots in the next growth season (e).

*Cardamine leucantha* (Tausch) O. E. Schulz [Brassicaceae] is an herbaceous plant that grows on the floor and margins of deciduous forests (Fig 2a and 2b). The species occurs in East Asia, and from Kyushu to Hokkaido in Japan. One of its conspicuous features is its clonal propagation through underground stolons that can grow more than 30 cm in length (Fig 2c). Sometimes a mother ramet produces two or more stolons simultaneously, and new ramets are formed at the tips (Fig 2d). The aboveground parts of mother ramets die at the end of each season and daughter ramets appear above ground the next year (Fig 2e). Daughter ramets often become disconnected from mother plants within 1–2 years. Therefore, a single genet of *C*. *leucantha* can develop into a group of disconnected ramets, spreading over tens of square meters or more.

The present study was conducted in a population located in a cool-temperate deciduous forest along the Toshibetsu River in Rikubetsu, Hokkaido, Japan (43°27′N, 143°46′E; 250 m a.s.l.). The forest is dominated by *Salix sachalinensis*; other common tree species are *Fraxinus mandshurica*, *Quercus crispula*, and *Ulmus davidiana*. A continuous population of *C*. *leucantha* extends over 3 ha along a stream, which allowed us to set up a large plot for conducting spatial analyses. At this site, *C*. *leucantha* ramets had elongated, upright stems which were 30–60 cm in length and produced inflorescences with white, self-incompatible, insect-pollinated flowers in June (Fig 2a). Seeds were dispersed from dehisced fruits in July. Forest floor herbs and ferns that are large enough to shade *C*. *leucantha* ramets included *Urtica thunbergiana*, *Cacalia hastata* subsp. *orientalis*, *Carex pilosa*, and *Dryopteris crassirhizoma*. The field study was conducted with the approval by Rikubetsu town office. This study did not involve any endangered or protected species.

### Sampling and environmental heterogeneity

**Fig 3:**
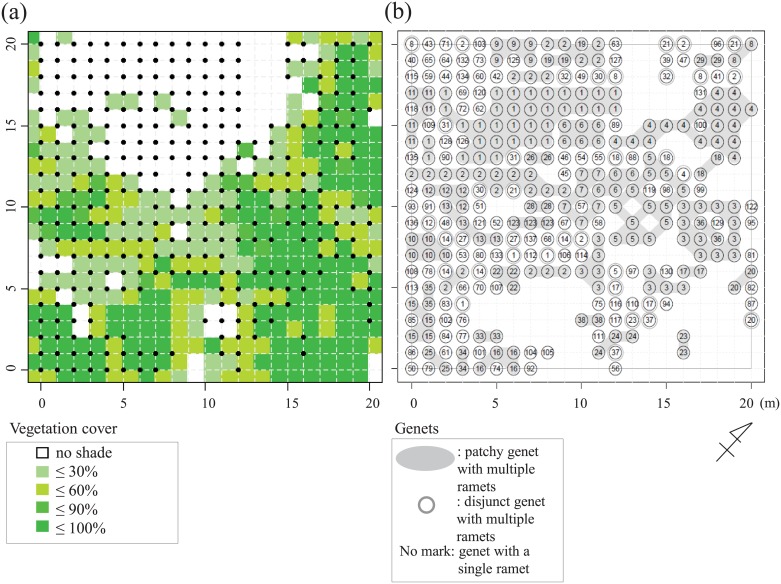
Sampling design. The study plot measured 20 m × 20 m. Vegetation cover data was used as a measure of environmental heterogeneity (a), and the spatial distribution of genets (groups of clonal ramets with shared genotypes) was determined through simple sequence repeat (SSR) analyses (b). In (a), the focal study plot is indicated by red lines. It consisted of four hundred 1-m^2^ quadrats. The vegetation cover of the forest floor (the fraction of the area covered by forest floor herbs and ferns large enough to shade *C*. *leucantha* ramets) was classified according to five categories of shading (no shade, ≤ 30%, ≤ 60%, ≤ 90%, or ≤ 100% vegetation cover) for each of the 484 quadrats (the focal 400 plus the surrounding 84). Black dots represent the sampling points. In (b), numbers represent genets, which are numbered in decreasing order of the number of ramets they contained. Patchy and disjunct genet members that belonged to genets with multiple ramets are grouped by shading and circles, respectively. Numbers without shading or circling represent unique genets found in only one sample.

We set up a 20 m × 20 m study plot divided by grid lines at 1-m intervals (Fig 3a) in the *C*. *leucantha* population. To represent environmental heterogeneity, we recorded the vegetation cover of the forest floor in each of 484 1-m^2^ quadrats separated by grid lines. These included 400 focal quadrats within the plot and 84 in the surrounding area. We defined vegetation cover as the fraction of the area covered by forest floor herbs and ferns large enough to shade *C*. *leucantha* ramets, and classified it into one of five categories: no shade; ≤ 30%; ≤ 60%; ≤ 90%; and ≤ 100% coverage. We also recorded the number of flowering ramets of *C*. *leucantha* for each quadrat.

In June 2010, we collected fully expanded fresh leaves on the top of one ramet at each of the 441 crossing points of the grid lines in the study plot (hereafter, grid points). At each grid point, we selected the ramet located nearest to the point for sampling, unless no ramets were present within 0.5 m of the grid point, in which case no leaves were collected for that grid point.

### DNA extraction

Sampled leaves were kept and dried in silica gel until DNA extraction. Genomic DNA was extracted using the cetyltrimethyl ammonium bromide method. The extracted DNA was dissolved in 20 μl of Tris ethylenediaminetetraacetic acid buffer (TE; 1 mM Tris-HCl, 0.1 mM EDTA, pH 8). DNA was extracted from all samples and stored at –20°C until analysis.

### SSR analysis

We amplified 5 ng of extracted DNA using a set of eight labeled primer pairs targeting highly polymorphic DNA microsatellite loci, according to a previously reported protocol (GenBank accession numbers are given in the reference) [46]. We performed size separation of the polymerase chain reaction (PCR) products using capillary electrophoresis on an ABI PRISM 3130 genetic analyzer (Applied Biosystems, Foster City, CA, USA). Semi-automated size scoring of banding patterns and genotyping was done using GENEMAPPER 3.7 (Applied Biosystems). We calculated the likelihood of errors in falsely ascribing genotypes to the same genet, *P*_gen_ [47], to be < 0.001 with the utilized primers for all samples. Thus, when all multilocus genotypes were shared by different ramets, we assumed that they belonged to a single genet.

### MS-AFLP analysis

MS-AFLP analyses were conducted using a modification of the original AFLP analysis protocol, with the enzyme combinations *EcoR*I-*Hpa*II and *EcoR*I-*Msp*I [48] (see S1 Appendix for the detailed protocol). We compared the MS-AFLP results for every sample to identify polymorphic epigenetic loci. *Hpa*II and *Msp*I cut DNA sequences at the same tetra-nucleotides (5′-CCGG-3′), but have different sensitivities to cytosine methylation at the restriction site. This method therefore allows determination of the methylation status of anonymous regions of the genome that are susceptible to methylation. *Hpa*II is sensitive to methylation of internal cytosines on both strands, and *Msp*I is sensitive to hemi-methylation of external cytosines. *Hpa*II and *Msp*I, therefore, do not cut sites corresponding to internal and external cytosine methylations, respectively.

We treated fragments that showed different presence/absence patterns among samples as epigenetic loci. Using four primer pairs (S1 Table), we detected 93 clear fragment-producing CCGG sites, 24 of which were polymorphic. We use the term “loci” to refer these 24 epigenetically polymorphic CCGG sites (referred to as the 24 MS-AFLP loci). For a particular 5′-CCGG-3′ site detected with MS-AFLP, four states exist, represented by the presence of the corresponding fragments for both *Hpa*II and *Msp*I cuts, for *Msp*I cuts only, for *Hpa*II cuts only, and for neither *Hpa*II nor *Msp*I cuts [49]. The presence of fragments for both cuts (condition I) infers that the corresponding 5′-CCGG-3′ site is non-methylated. The presence of a fragment in the *EcoR*I-*Msp*I digestion and its absence in the *EcoR*I-*Hpa*II digestion (condition II) was attributed to methylation on the internal cytosines. The presence of a fragment only in the *EcoR*I-*Hpa*II digestion (condition III) was interpreted as methylation of the external cytosine. Notably, the absence of fragments for both cuts (condition IV) represents either methylation of both (internal and external) cytosines or loss of the digestion site via mutation [45, 50, 51]. Depending on the treatment of the condition IV, different scoring approaches have been applied in previous MS-AFLP analyses (reviewed in [45]).

We applied the mixed scoring methods for MS-AFLP analysis [45, 52, 53]. In this approach, by using the information of conditions I, II and III, three subepiloci were generated from each of 24 MS-AFLP locus, i.e., coding states of the non-methylated (n-subepiloci), the CG-methylated (m-subepiloci) and the CHG-hemimethylated (h-subepiloci). Furthermore, for three MS-AFLP loci in which sequences were successfully determined (S2 Table), we sequenced the digestion sites for selected samples with condition IV (44 including 37 genet × three locus combinations) and confirmed that sequence polymorphism was absent (S3 Table; see below for primer designing and locus selection).

We determined epigenetic state scoring error rates for each primer combination using repeated analyses of 11 leaf samples. We estimated rates as the percentage of mismatch between replicates (S1 Table). For the polymorphic MS-AFLP sites, average scoring error rates were 3.9% for *Eco*RI-*Hpa*II analyses and 7.4% for *Eco*RI-*Msp*I analyses (for all primer pairs; see S2 Table). We further determined the sequences of the analyzed MS-AFLP loci using selective restriction fragment amplifications (S2 Appendix) to confirm that our procedure successfully selected 5′-CCGG-3′ sites from the *C*. *leucantha* genome. Because *C*. *leucantha* is a member of Brassicaceae, we expected that we would find similar sequences on the reported genomes of closely related *Brassica* species and *A*. *thaliana*. Five fragments were successfully sequenced and annotated based on sequence similarity using BLAST (Basic Local Alignment Search Tool) on the DDBJ (DNA Data Bank of Japan). All five sequences showed similarity to parts of the reported sequences of close relatives in *Arabidopsis* and *Brassica* (S2 Table), so we judged that our procedures were working correctly. We further successfully designed primers to determine sequences of digested sites for three of above five MS-AFLP loci by mapping the corresponding fragment sequences to the *Arabidopsis* and *Brassica* genome. Using these primers, sequences of the three MS-AFLP loci were determined for selected samples to evaluate the occurrence of mutations at the digestion site (described above).

#### Stability of epigenetic variation

In the above MS-AFLP analysis, we used a single leaf from the top of each ramet, all of which were collected on the same day (16 June 2010). In contrast to genotypes, however, epigenetic states may change depending on leaf position and season. To evaluate the stability of the epigenetic patterns detected with MS-AFLP analyses in this study, we performed an additional experiment comparing MS-AFLP patterns between leaves from different positions and in different seasons.

To assess the positional stability of the epigenetic state, we analyzed the fragments at *EcoR*I-*Msp*I and *EcoR*I-*Hpa*II digestion sites of 24 polymorphic MS-AFLP loci in all leaves (three, six, eight, and 13, respectively) for four randomly selected ramets. We calculated the positional stability of each MS-AFLP site as the percentage of all leaves from each ramet that shared the MS-AFLP pattern of the top leaf and averaged them across the four ramets.

To assess the seasonal stability of the epigenetic state, we sampled the second leaf from the top of 11 ramets in September, and compared their MS-AFLP patterns with those of the top leaf from the same ramet collected in June. The probability of matching was calculated across the 11 ramets for each of the 24 MS-AFLP loci. Stability was calculated as the ratio of samples showing the same pattern of methylation to all samples.

### Data analysis

We performed spatial autocorrelation analyses on all ramets for genetic variation based on genotypes and for epigenetic variation based on epigenotypes using the software package GenAlEx version 6 [54]. Multi-locus epigenotypes were determined by epigenetic states (methylated or non-methylated) across all examined epigenetic loci. The autocorrelation coefficient *r* was generated between pairwise geographical and pairwise squared genotypic or epigenotypic distance matrices (a Euclidian distance metric) for 1-m distance classes up to 20 m. The coefficient *r* provides a measure of the genetic/epigenetic similarity between pairs of samples with a geographical separation within a specified distance class. Distance classes were chosen to represent an even number of pairwise comparisons. We tested the statistical significance of *r* for each distance class, using random permutations and 9,999 bootstrapping cycles.

As described in the conceptual framework section, we used a hierarchical Bayesian approach to quantify the effects of genet, vegetation cover, and other spatial factors on probability of having score 1 for the three subepiloci of 24 MS-AFLP polymorphic loci. The probability for MS-AFLP locus *i* in sample *j* for genet *k*, i.e., *q*_*ijk*_, is defined using a combination of the logit link function and a linear predictor, *logit* (*q*_*ijk*_) = *g*_0_ + *g*_*i*_ + *g*_*ik*_ + (*e*_0_ + *e*_*i*_)*x*_*j*_ + *s*_*j*_. To estimate the posterior distributions for all parameters, we selected their prior distributions as follows. Gaussian non-informative priors were specified for *g*_0_ and *e*_0_ because they were common to all sampling points. Gaussian hierarchical priors were specified for the other parameters because they represented the random effects of MS-AFLP locus *i* and genet *k*. The prior of parameter *s*_*j*_, which represents the spatial random effects at sampling point *j*, was the Gaussian distribution of the mean, *μ*_*j*_, embedded in the conditional auto-regressive model [55], in which *μ*_*j*_ is equal to the mean of {*μ*_*j′*_} for all adjacent spatial blocks of *j*. All dispersion parameters for Gaussian hierarchical priors were specified as uniform distributions that ranged from 0–100. We used the MCMC method to sample from the posterior distributions of the above parameters in JAGS 4.2.0 [56]. We obtained posterior samples from three independent Markov chains in which 1,000 × 3 values were sampled, with a 50-iteration interval, after a burn-in of 1,000 iterations. The convergence of the Markov chains was evaluated in R [57] by comparing the variance within each chain and among the chains for each parameter. JAGS code for analysis was written in S3 Appendix.

## Results

### Environmental heterogeneity and ramet distribution

With respect to environmental heterogeneity, as represented by vegetation cover, there were 119, 74, 67, 19, and 121 quadrats with 0, ≤ 30%, ≤ 60%, ≤ 90%, and ≤ 100% vegetation cover, respectively (Fig 3a). There was a spatial cline in vegetation cover across the study site, with vegetation becoming denser toward the southeast and southwest (Fig 3a). We found flowering *C*. *leucantha* ramets in 68% (251/400) of the 1 m × 1 m focal quadrats within the study plot. Altogether, 1,244 flowering ramets were counted within the plot, at a density of 1–33 ramets/m^2^ (3.1 ± 4.8, mean ± SD). Vegetation cover and number of flowering ramets were negatively correlated (Spearman rank correlation, *r* = –0.29, *p* < 0.001).

In total, 335 leaves from 176 flowering and 159 vegetative ramets were sampled at the grid points (intersections of grid lines / quadrat boundaries) and no ramets were found at 106 grid points (sampled grid points are shown as dots in Fig 3a). The growth stage of the sampling ramets (flowering or vegetative) had no effect on the results of the SSR or MS-AFLP analyses.

### Genet distribution

**Fig 4:**
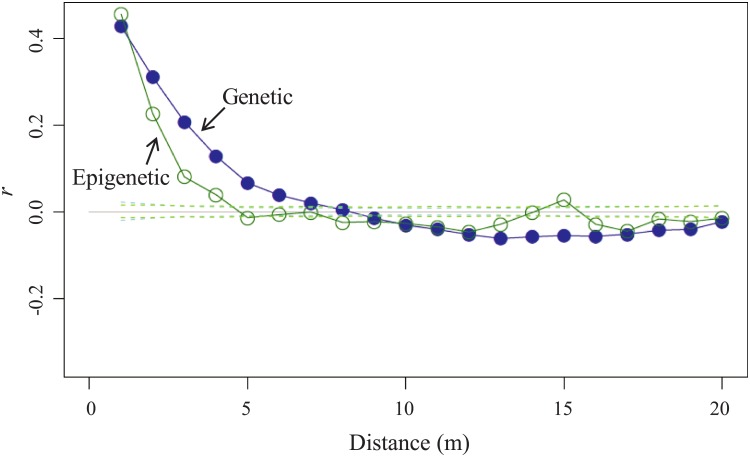
Correlograms (solid lines) calculated for genetic SSR (blue) and epigenetic MS-AFLP (green) markers. In the spatial autocorrelation analyses, the correlation coefficient (*r*) was calculated using ramet combinations for each distance class set at 1-m intervals. All eight SSR loci and the 24 MS-AFLP loci were used and all ramets were included in the analysis. 95% confidence intervals are denoted for genetic markers (dotted line) and epigenetic markers (dashed line) in corresponding colors.

Multilocus genotypes for eight SSR loci were successfully determined for 332 ramets, and we identified 137 multilocus genotypes (Fig 3b). Assuming that ramets with a shared multilocus genotype were members of a single genet, the sampled ramets were composed of six large genets comprising 10 or more ramets, 48 smaller genets comprising < 10 multiple ramets, and 83 unique genets were comprised of only a single ramet each (Fig 3b and S1 Fig). We numbered these genets in decreasing order of the number of ramets they contained, designating the largest one as genet 1. Genets were patchily distributed, and the largest genet (genet 1) was identified in 37 ramets, followed by the second largest genet (genet 2) in 29 ramets (Fig 3b). Spatial autocorrelation analysis on all ramets detected significant aggregation of shared multilocus genotypes within a distance of up to 7 m (Fig 4).

### Spatial epigenetic variation and genet-specific probabilities

**Fig 5:**
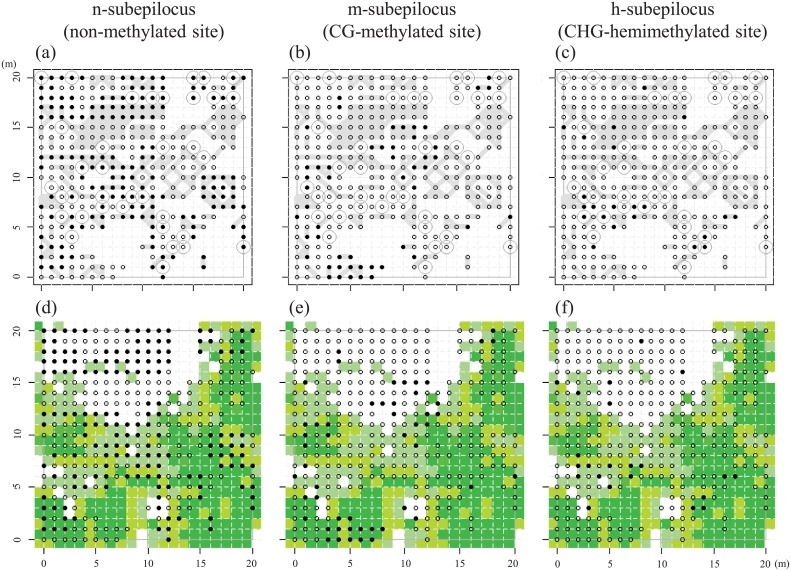
Spatial distributions of epigenetic status for the n-subepiloci (non-methylated site) [both cut (condition I); (a, d)], m-subepiloci (CG-methylated site) ([EcoRI-HpaII digestion; (b, e)], and h-subepiloci (CHG-hemimethylated site) [EcoRI-MspI digestion; (c, f)] of an MS-AFLP locus, Lo3-343, plotted on a map of genets (a, b, c) and vegetation cover (d, e, f). Open and closed circles represent 0 and 1 scores, respectively. Genet distribution and vegetation cover as for Fig 3.

We analyzed 24 MS-AFLP polymorphic loci for all 332 ramets for which SSR genotypes were determined. The global probabilities of the 24 MS-AFLP loci averaged 12.9% (range: 0%–91.6%), 68.6% (range: 7.2%–98.2%), and 0.7% (range: 0%–6.6%) for n-subepiloci, m-subepiloci and h-subepiloci, respectively (Table 1). We observed patchy distribution of epigenetic patterns for some MS-AFLP loci (Fig 5; and see S2 Fig for the maps of the 24 MS-AFLP loci), and spatial autocorrelation analyses across the 24 MS-AFLP loci showed significant aggregation of overall methylation patterns within a distance of up to 4 m (Fig 4).

**Table 1:** Global probabilities and numbers of genets whose genet-specific methylation probability deviated from global probabilities^a^.

Locus	Global probability (methylation rate %)			Genet-specific probability (no. of genets with different probabilities)					
	n-subepiloci	m-subepiloci	h-subepiloci	n-methyl		m-methyl		h-methyl	
				Low	High	Low	High	Low	High
**Lo1-042**	5.4	67.5	0.6	0	3	8	0	0	0
**Lo1-080**	3.0	95.5	0.0	0	2	2	0	0	0
**Lo1-123**	2.7	93.7	0.6	0	2	3	0	0	0
**Lo1-193**	1.2	87.7	0.0	0	1	6	0	0	0
**Lo1-203**	0.0	29.5	0.6	0	0	8	2	0	0
**Lo1-225**	0.3	82.5	0.3	0	1	5	0	0	0
**Lo2-147**	1.8	13.0	1.5	0	1	0	6	0	0
**Lo2-170**	16.9	82.5	0.0	0	1	2	0	0	0
**Lo2-181**	0.6	80.7	0.6	0	2	6	0	0	0
**Lo2-184**	1.5	68.7	0.6	0	1	2	2	0	0
**Lo2-265**	5.1	92.2	0.3	0	1	3	0	0	0
**Lo2-292**	24.7	25.6	2.1	2	10	1	7	0	0
**Lo3-082**	16.9	81.9	0.0	0	6	7	0	0	0
**Lo3-096**	70.2	28.3	1.2	7	2	1	8	0	0
**Lo3-100**	1.2	97.0	0.0	0	2	3	0	0	0
**Lo3-165**	14.5	84.3	0.3	0	6	6	0	0	0
**Lo3-257**	1.2	95.2	0.6	0	1	1	0	0	0
**Lo3-300**	91.6	7.2	0.3	4	0	0	4	0	0
**Lo3-325**	0.0	98.2	0.0	0	0	2	0	0	0
**Lo3-343**	48.8	15.7	6.6	4	6	0	7	0	0
**Lo4-075**	0.9	57.8	0.3	0	1	9	4	0	0
**Lo4-091**	0.6	92.5	0.6	0	1	2	0	0	0
**Lo4-147**	0.0	94.0	0.6	0	0	4	0	0	0
**Lo4-235**	0.3	74.4	0.0	0	1	6	0	0	0

^a^ The number of significantly higher- or lower-methylated genets is listed for the 24 MS-AFLP loci examined (methylation-subepiloci, m-subepiloci, and h-subepiloci of 24 epigenetic loci).

**Fig 6:**
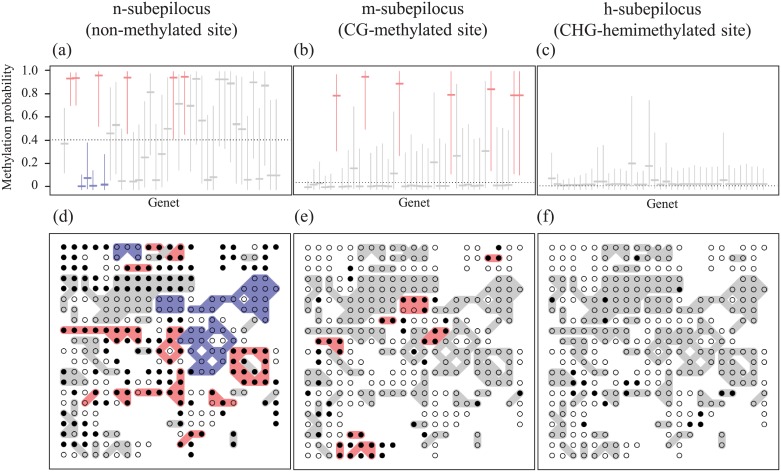
Genet-specific probability (a–c) and spatial distribution of genet-specific methylation status (d–f) for the n-subepiloci (a, d), m-subepiloci (b, e) and h-subepiloci (f, g) of the MS-AFLP locus, Lo3-343. In a–c, medians and 95% BCI of genet specific probabilities are indicated by horizontal lines and vertical lines, respectively. Genets are arranged from left to right in descending order of genet size, i.e. number of ramets, in each diagram. Horizontal dotted lines indicate the median of global probabilities. In the maps (d–f), open and closed circles represent 0 and 1 scores, respectively. Patchy genet members that belong to genets with multiple ramets are grouped by shading. Genets with significantly higher and lower genet-specific probabilities than the global probability (no overlap between 95% BCI of genet-specific probability and median of global probability) are shown in red and blue, respectively, in all panels.

The predominant effect of genet distribution was apparent in the spatial patterns of epigenetic variation. The hierarchical Bayesian model allowed us to evaluate genet-specific effects on methylation probability in the presence of random effects (see positive sigma terms in locus, cover, genet, and special heterogeneity of three subepiloci in S4 Table). The global probabilities in n-subepiloci were generally low except for three loci (Lo3-096, Lo3-300, and Lo3-343, Table 1). The probabilities varied largely in m-subepiloci, while they were extremely low in all h-subepiloci (Table 1). The deviations of genet-specific probabilities were detected in 21 n-subepiloci (S3a Fig) and in all m-subepiloci (S3b Fig). One to 12 genets for n-subepiloci and one to 13 genets showed lower or higher genet-specific probabilities (Table 1). For n-subepilocus at the locus Lo3-343, for example, four and six genets showed genet-specific probabilities that were significantly lower (negatively deviated 95% BCI, shown in blue in Fig 6a and 6d) and higher (positively deviated 95% BCI, shown in red in Fig 6a and 6d), respectively, than the global probability (estimated as 57.8%, Table 1). For m-subepilocus at the same MS-AFLP locus, seven genets showed significantly higher genet-specific probabilities (Fig 6b and 6e), and no genet showed higher and lower local probability for h-subepilocus (Fig 6c and 6f).

**Fig 7:**
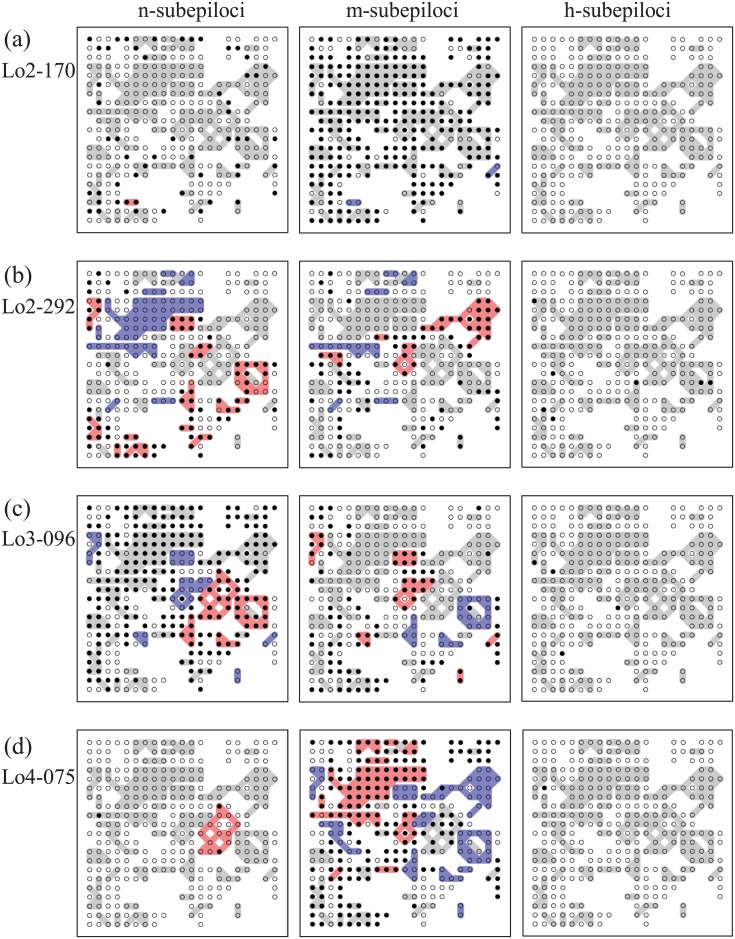
Spatial distributions of genet-specific methylation status of four selected loci: Lo2-170 (a), Lo2-292 (b), Lo3-096 (c), and Lo4-075 (d). Three diagrams for each locus represent n-subepiloci (left), m-subepiloci (middle) and h-subepiloci (right). Symbols as for Figs 1 and 6. For statistical details, see the corresponding probability deviance diagrams in S3 Fig.

In m-subepiloci of 24 MS-AFLP loci, significant genet-specific methylation probabilities were detected at many genets (Fig 7), e.g. eight, nine, and 13 genets for Lo2-292, Lo3-096, and Lo4-075 (Fig 7b–7d), respectively. In addition, significant genet-specific patterns were also represented n-subepiloci (Fig 7). In these examples, based on the model analysis, frequent changes in methylation patterns at genet boundaries are to be expected. In contrast, for m-subepiloci at the locus Lo2-170, one-scored ramets were randomly scattered on the map, suggesting a weak genet contribution (Fig 7a). Methylation polymorphisms were quite low in h-subepiloci and therefore no genet-specific methylation probabilities were found (Table 1; Fig 7).

In the largest genet (genet 1), methylation rates for m-subepiloci were significantly lower and higher compared to the global probability at one and two MS-AFLP loci, respectively (S2 and S3 Figs). In a genet with the highest number of MS-AFLP loci that differed from the global probability (genet 33), two and six sites displayed significantly higher and lower methylation probabilitiesat m-subepiloci, respectively. In five genets (genets 12, 30, 34, 35, and 36), no MS-AFLP loci showed significantly different genet-specific local probabilities.

Results of posterior distributions indicated that vegetation cover had weak effects in determining methylation probabilities for all 24 MS-AFLP loci except for n-subepiloci of Lo2-292 and m-subepiloci of Lo2-184 (S4 Table). In these loci, negative relationships between methylation probability and vegetation cover were detected (S4a and S4b Table; Fig 5)

### Stability of epigenetic variation

Positional stabilities ranged from 78.1% (Lo3-100 for CG-methylation) to 100% (Table 2), and averaged 94.6% across the 24 MS-AFLP loci. Some MS-AFLP loci tended to show a different MS-AFLP pattern in the lowest leaves, probably as a result of leaf senescence. Seasonal stability for each locus ranged from 41.7% (Lo2-184 for CHG-hemimethylation) to 100% (Table 2) and averaged 83.7% across the 24 MS-AFLP loci. This indicates that the epigenetic patterns detected in this study were relatively robust against changes in leaf-position and date, as long as the samples were taken from the same ramets within a single growth season.

**Table 2:** Positional and seasonal stabilities of methylation (concordance rates of MS-AFLP patterns)^a^.

Locus	Leaf position^b^		Seasonal changes^c^	
	CG site	CHG site	CG site	CHG site
**Lo1-042**	92.7	96.9	66.7	58.3
**Lo1-080**	100	93.8	91.7	66.7
**Lo1-123**	100	100	75.0	58.3
**Lo1-193**	95.8	96.9	91.7	66.7
**Lo1-203**	100	100	100	91.7
**Lo1-225**	100	95.8	91.7	66.7
**Lo2-147**	93.9	100	83.3	91.7
**Lo2-170**	98.1	100	100	100
**Lo2-181**	98.1	96.9	100	100
**Lo2-184**	98.1	100	41.7	75.0
**Lo2-265**	86.6	90.6	75.0	83.3
**Lo2-292**	100	96.9	75.0	83.3
**Lo3-082**	86.5	100	83.3	100
**Lo3-096**	88.0	100	100	100
**Lo3-100**	83.7	78.1	100	100
**Lo3-165**	94.2	86.6	100	66.7
**Lo3-257**	100	90.6	100	83.3
**Lo3-300**	82.3	87.5	66.7	91.7
**Lo3-325**	100	88.5	66.7	83.3
**Lo3-343**	91.7	90.6	91.7	91.7
**Lo4-075**	90.6	100	100	91.7
**Lo4-091**	93.8	96.9	83.3	58.3
**Lo4-147**	90.6	91.7	83.3	83.3
**Lo4-235**	100	96.9	83.3	75.0

^a^ Analyzed using MS-AFLP across leaf positions and seasons.

^b^ The ratio of leaves that had identical methylation patterns to the top leaf. The values are the averages of four randomly selected ramets with three, six, eight or 13 leaves per ramet, respectively.

^c^ The ratio of leaves that had identical methylation patterns between June and September. Values are averages of 11 ramets.

## Discussion

Spatial structures of genetic variation show long-lasting patterns. These are major components of the ecology and evolution of sessile organisms such as plants, fungi, and some invertebrate animals [1, 3, 58]. In clonal organisms that exhibit hierarchical individuality, groups of genetically identical but physiologically independent individuals spread across heterogeneous environments in the habitat. One expects to find a specific spatial structure of epigenetic variation in sessile organisms, depending on habitat heterogeneity and genetic structures of the populations under investigation, because epigenetic modification of DNA sequences is under the control of both genetic and environmental factors. By studying a population of the clonal plant *C*. *leucantha*, we showed that a distinct spatial structure in the variation of DNA cytosine methylation patterns existed in the natural plant population.

For clonal plant populations in spatially heterogeneous environments, each ramet is influenced by a unique combination of its genetic background and environment. We therefore considered the observed spatial pattern to be an epigenetic consequence ensuing from the interface between genetic and environmental controls. The distributions of epigenetic variation were non-random, and primarily took the form of aggregations of shared epigenetic states. Our spatial autocorrelation analysis showed significant aggregations within a distance of 4 m for the MS-AFLP markers and 7 m for the SSR markers (Fig 4). However, comparing spatial scales derived from SSR and AFLP-based methods requires caution [59, 60]. In our study, epigenetic variation was largely attributable to the patchy genet distribution. Genet boundaries were, therefore, likely to set an upper limit on the spatial size of epigenetic aggregations. The term “genetic structuring” was coined to refer to spatial aggregations of individuals with shared genetic variation, based on accumulating studies on the spatial distribution of genetic variation in natural organismal populations [61–63]. We therefore use the term “epigenetic structuring” to refer to the spatial aggregation of epigenetic variation within populations.

Among the various determinants of the spatial distribution of epigenetic variation, the effects of genets and environment must be evaluated for each epigenetic locus. Our study demonstrates that hierarchical Bayesian modeling can serve as a reliable tool for the analysis of spatial epigenetic structures in clonal plant populations. It allows us to handle the probabilistic nature of epigenetic status in the complex natural context where genets and environmental factors are unevenly distributed across space.

Overall, both global methylation probabilities and spatial patterns of genet-specific probabilities varied across MS-AFLP sites. Although global probabilities showed substantial fluctuations across MS-AFLP sites, we found a general tendency for the probabilities of methylated ramets to be high for the CG methylations and low for the CHG methylations. In the mixed scores, the patterns were reflected as pronounced methylation variation in m-subepiloci and low variation in h-subepiloci (see S4 Table for details). These patterns correspond well to known mechanisms of methylation maintenance at CG and CHG cytosines. Existing internal cytosine methylation at a particular 5′-CCGG-3′ can be transmitted into a newly formed complementary DNA sequence via cell division [64, 65]. In contrast, *de*-*novo* methylation predominates for external cytosines [66]. Previous studies that have quantified MS-AFLP variation in plants have also reported similar patterns [30, 50].

Genet-specific differences in methylation probabilities were found in all of the 24 MS-AFLP loci examined (i.e. at least m-episubloci). Therefore, differences in methylation patterns between genets may be attributable to differences in the genetic backgrounds of the genets. Genetic controls of methylation polymorphisms have been reported in *A*. *thaliana* accessions (e.g. [29]). In natural populations, however, genet-specific effects may also be attributable to the demographic profile of genets, including the time elapsed since their establishment, clonal expansion, and other characteristics [15, 67, 68]. In our analyses, genet specific-effects were detected in the form of probabilistic deviance from the global probabilities. Because global probability was determined by the composition of ramets from different genets, it may be expected to be determined by the epigenetic status of dominant genets. In our study, genet size (number of ramets per genet) which reflected dominance in the population, did not explain the methylation patterns. The largest three genets often showed genet-specific methylation probabilities that were distinct from the global probabilities.

Local microenvironments are another potential determinant of epigenetic variation. Environmental stresses such as temperature, salinity, and ultraviolet light have been reported to change the epigenetic status of plants [41, 69–71]. In this study, vegetation cover was negatively correlated with the number of flowering ramets per unit area. However, we detected effects of vegetation cover for only two sites in a single MS-AFLP locus. More detailed measurements of microenvironments or studies of populations with steeper environmental gradients may allow the detection of environmental effects on the spatial patterns of epigenetic variation in natural populations.

Another possible explanation is that environmental effects on epigenetic status may be localized in the genome at the locations of environment-responsive genes. Hence, we may have a lower chance of detecting environmental effects using methods such as MS-AFLP, which are based on the random sampling of epigenetic loci from the genome. Nonetheless, using the MS-AFLP method, Lira-Medeiros *et al*. [15] detected different methylation patterns between two natural mangrove populations that grow in contrasting salt environments but display no genetic differentiation in SSR markers. It remains to be determined whether genome-wide differences in epigenetic status between populations involves genetic differentiation in a specific region on the genome that can be maintained by natural selection, even under extensive gene flow. Future investigations should address this by examining epigenetic variation for the specific genome region(s) involved in the responses to each environmental factor.

Although laboratory experiments are a strong approach for identifying the causal factors that affect epigenetic variation, it is necessary to accumulate knowledge on the distribution of epigenetic variation in natural populations before we can comprehensively understand the role of epigenetics. Emerging evidence suggests that some epigenetic variation, including cytosine methylation patterns, comprises a component of fitness variation among individuals in natural plant populations [24, 26, 72–74]. Herrera & Bazaga [75] showed that the intensity of herbivory alters cytosine methylation patterns among individuals in wild populations of *Viola cazorlensis*. Furthermore, some of the methylation patterns are likely to be passed on from mother to daughter ramets via vegetative propagation. For example, the DNA methylation changes induced by environmental stresses are faithfully transmitted to offspring in apomictic dandelions [18]. In another example, the methylation patterns of meristem-issued rejuvenated plants did not differ from that of the mother tree in three clonal lines of the giant sequoia *Sequoiadendron giganteum* after seven years [68]. Epigenetic mechanisms may therefore allow clonal plants to respond conservatively to environmental changes by referring to environments experienced by the clonal lineages [76]. One direction for future research would be the spatial analysis of epigenetic variation in which functional roles have already been identified, especially in ecologically important traits.

## Supporting information

S1 Table Primer combinations used, number of markers (loci) in the size range 45–450 bp, and scoring error rates. (DOCX)

S2 Table Top hit homologous sequences of five MS-AFLP polymorphic loci. The sequences were annotated based on sequence similarity using a BLAST search of the DDBJ.
(DOCX)

S3 Table Sequences of representative samples identified as condition IV (uncut by both enzymes) at three MS-AFLP loci. For the locus Lo2-147, four samples with conditions I, II and III were included for comparisons.
(DOCX)

S4 Table Summary of posterior distributions in the hierarchical Bayesian model for the n-subepiloci (a), m-subepiloci (b) and h-subepiloci (c) of the 24 polymorphic MS-AFLP loci. (DOCX)

S1 Fig Frequency distribution of the number of ramets per genet. (PPT)

S2 Fig Spatial distribution of genet-specific methylation statuses for the n-subepiloci (a), m-subepiloci (b) and h-subepiloci (c) of the 24 polymorphic MS-AFLP loci. Detailed explanations are provided in Figs 1 and 6.
(PDF)

S3 Fig Genet-specific methylation probabilities for the n-subepiloci (a), m-subepiloci (b) and h-subepiloci (c) of the 24 polymorphic MS-AFLP loci. Detailed explanations are provided in Figs 1 and 6.
(PDF)

S1 Appendix MS-AFLP protocol. (DOCX)

S2 Appendix Sequencing of MS-AFLP loci. (DOCX)

S3 Appendix JAGS code. (DOCX)
