# Supplementary material for: Genet-specific DNA methylation probabilities detected in a spatial epigenetic analysis of a clonal plant population
Source: PLoS One. 2017 May 22;12(5):e0178145. doi: 10.1371/journal.pone.0178145 (PMC5439711; doi:10.1371/journal.pone.0178145)
Supplement: S1 Table — (DOCX) [file pone.0178145.s001.docx]

**S1 Table.** **Primer combinations used, number of MS-AFLP loci in the size range of 45-450 bp, and scoring error rates.**

|  | Total  MS-AFLP loci^a^ | Scoring error rate (%)^b^ | |
| --- | --- | --- | --- |
| Primer combination |  | CG site (*Hpa*II) | CHG site (*Msp*I) |
| *EcoR*I + AC/*Hpa*II-*Msp*I + CG | 6 (31) | 1.4 (1.6) | 2.9 (3.5) |
| *EcoR*I + CG/*Hpa*II-*Msp*I + TTA | 6 (19) | 6.7 (4.7) | 10.6 (10.5) |
| *EcoR*I + AC/*Hpa*II-*Msp*I + TTA | 8 (22) | 4.4 (4.5) | 6.7 (5.9) |
| *EcoR*I + CT/*Hpa*II-*Msp*I + AGG | 4 (21) | 2.5 (2.9) | 11.4 (5.6) |
| All combined | 24 (93) | 3.8 (3.2) | 7.4 (6.1) |

^a^ Number of polymorphic methylation markers detected in 332 samples in this study (followed by the total including monomorphic markers in parentheses).

^b^ Calculated from the 11 individual plants that were reassayed as 100 × (number of discordant scores in two independent analyses) / (number of scored markers × number of individuals).
