# Supplementary material for: Genet-specific DNA methylation probabilities detected in a spatial epigenetic analysis of a clonal plant population
Source: PLoS One. 2017 May 22;12(5):e0178145. doi: 10.1371/journal.pone.0178145 (PMC5439711; doi:10.1371/journal.pone.0178145)
Supplement: S2 Table — The sequences were annotated based on sequence similarity using a BLAST search of the DDBJ. (DOCX) [file pone.0178145.s002.docx]

**S2 Table. Top hit homologous sequences of five MS-AFLP polymorphic loci. The sequences were annotated based on sequence similarity using a BLAST search of the DDBJ.**

| Locus | Seq. length (bp) | Accession | Description | E-value | Identities |
| --- | --- | --- | --- | --- | --- |
| Lo1-123 | 98 | CP002684.1 | *Arabidopsis thaliana* chromosome 1, complete sequence | 7.00E-17 | 80/98 (82%) |
|  |  | NM_148435.1 | *Arabidopsis thaliana* CLAVATA3/ESR-related protein 20 (CLE20) mRNA, complete cds | 7.00E-17 | 80/98 (82%) |
|  |  | AC005322.2 | *Arabidopsis thaliana* chromosome 1 BAC T7A14 sequence, complete sequence | 7.00E-17 | 80/98 (82%) |
| Lo2-147 | 144 | AB005248.1 | *Arabidopsis thaliana* genomic DNA, chromosome 5, P1 clone:MXI10 | 8.00E-25 | 112/142 (79%) |
| Lo2-170 | 146 | AB180901.1 | *Brassica oleracea* S-12 SRK gene for S-locus receptor kinase, complete cds | 7.00E-06 | 55/64 (85%) |
|  |  | AB180903.2 | *Brassica oleracea* S-15 SRK gene for S-locus receptor kinase, complete cds | 7.00E-06 | 55/64 (85%) |
| Lo2-265 | 242 | BT005418 | *Arabidopsis thaliana* clone U50382 unknown protein (At4g02425) mRNA,complete cds. | 5.00E-70 | 181/196 (92%) |
|  |  | AL161494 | *Arabidopsis thaliana* DNA chromosome 4, contig fragment No. 6. | 5.00E-70 | 181/196 (92%) |
|  |  | AK117597 | *Arabidopsis thaliana* mRNA for unknown protein, complete cds, clone: RAFL17-24-H02. | 5.00E-70 | 181/196 (92%) |
| Lo3-82 | 64 | XM_002866914.1 | *Arabidopsis lyrata* subsp. *lyrata* hypothetical protein, mRNA | 1.00E-06 | 46/58 (79%) |
