# Supplementary material for: Genet-specific DNA methylation probabilities detected in a spatial epigenetic analysis of a clonal plant population
Source: PLoS One. 2017 May 22;12(5):e0178145. doi: 10.1371/journal.pone.0178145 (PMC5439711; doi:10.1371/journal.pone.0178145)
Supplement: S3 Table — For the locus Lo2-147, four samples with conditions I, II and III were included for comparisons. (DOCX) [file pone.0178145.s003.docx]

**S3 Table. Sequences of representative samples identified as condition IV (uncut by both enzymes) at three MS-AFLP loci.** For the locus Lo2-147, four samples with conditions I, II and III were included for comparisons.

|  |  | Methylation | | |  |
| --- | --- | --- | --- | --- | --- |
| Locus | Genotype | Condition^a^ | CG site^b^ | CHG site^b^ | Sequence |
| Lo1-123 | g3 | IV | 1 | 1 | CTCCGGCGCCGACCACCGACNNNNNNNATTGATCAGGGAGAAA |
| Lo1-123 | g4 | IV | 1 | 1 | CTCCGGCGCCGACCACCGACGGGGTAGATTGATCAGGGAGAAACTCTCTGTCTTCACC |
| Lo1-123 | g20 | IV | 1 | 1 | CTCCGGCGCCGACCACCGACGGGGTAGATTGATCAGGGAGAAACTCTCTGTCTTCACC |
| Lo1-123 | g20 | IV | 1 | 1 | CTCCGGCGCCGACCACCGACGGGGTAGATTGATCAGGGAGAAACTCTCTGTCTTCACC |
| Lo1-123 | g25 | IV | 1 | 1 | CTCCGGCGCCGACCACCGACGGGGTAGATTGATCAGGGAGAAACNNNNNNNCTTC |
| Lo1-123 | g99 | IV | 1 | 1 | CTCCGGCGCCGACCACCGACGGGGTAGATTGATCAGGGAGAAACNNNNNNNCTTC |
| Lo1-123 | g113 | IV | 1 | 1 | CTCCGGCGCCGACCACCGACGGGGTAGATTGATCAGGGAGAAACNNNNNNNCTTC |
| Lo2-147 | g1 | IV | 1 | 1 | AGAATTGCCTTGATTTCCCGGTTAGAAACTTCGACCTGCCCACTTGTTTGGGGATGATACGGTGTCGCAA |
| Lo2-147 | g1 | IV | 1 | 1 | AGAATTGCCTTGATTTCCCGGTTAGAAACTTCGACCTGCCCACTTGTTTGGGGATGATACGGTGTCGCAA |
| Lo2-147 | g2 | IV | 1 | 1 | AGAATTGCCTTGATTTCCCGGTTAGAAACTTCGACCTGCCCACTTGTTTGGGGATGATACGGTGTCGCAA |
| Lo2-147 | g2 | I | 0 | 0 | AGAATTGCCTTGATTTCCCGGTTAGAAACTTCGACCTGCCCACTTGTTTGGGGATGATACGGTGTCGCAA |
| Lo2-147 | g2 | IV | 1 | 1 | AGAATTGCCTTGATTTCCCGGTTAGAAACTTCGACCTGCCCACTTGTTTGGGGATGATACGGTGTCGCAA |
| Lo2-147 | g2 | IV | 1 | 1 | AGAATTGCCTTGATTTCCCGGTTAGAAACTTCGACCTGCCCACTTGTTTGGGGATGATACGGTGTCGCAA |
| Lo2-147 | g3 | IV | 1 | 1 | AGAATTGCCTTGATTTCCCGGTTAGAAACTTCGACCTGCCCACTTGTTTGGGGATGATACGGTGTCGCAA |
| Lo2-147 | g4 | IV | 1 | 1 | AGAATTGCCTTGATTTCCCGGTTAGAAACTTCGACCTGCCCACTTGTTTGGGGATGATACGGTGTCGCAA |
| Lo2-147 | g4 | IV | 1 | 1 | AGAATTGCCTTGATTTCCCGGTTAGAAACTTCGACCTGCCCACTTGTTTGGGGATGATACGGTGTCGCAA |
| Lo2-147 | g5 | IV | 1 | 1 | AGAATTGCCTTGATTTCCCGGTTAGAAACTTCGACCTGCCCACTTGTTTGGGGATGATACGGTGTCGCAA |
| Lo2-147 | g6 | IV | 1 | 1 | AGAATTGCCTTGATTTCCCGGTTAGAAACTTCGACCTGCCCACTTGTTTGGGGATGATACGGTGTCGCAA |
| Lo2-147 | g8 | IV | 1 | 1 | AGAATTGCCTTGATTTCCCGGTTAGAAACTTCGACCTGCCCACTTGTTTGGGGATGATACGGTGTCGCAA |
| Lo2-147 | g9 | IV | 1 | 1 | AGAATTGCCTTGATTTCCCGGTTAGAAACTTCGACCTGCCCACTTGTTTGGGGATGATACGGTGTCGCAA |
| Lo2-147 | g10 | IV | 1 | 1 | AGAATTGCCTTGATTTCCCGGTTAGAAACTTCGACCTGCCCACTTGTTTGGGGATGATACGGTGTCGCAA |
| Lo2-147 | g11 | IV | 1 | 1 | AGAATTGCCTTGATTTCCCGGTTAGAAACTTCGACCTGCCCACTTGTTTGGGGATGATACGGTGTCGCAA |
| Lo2-147 | g12 | IV | 1 | 1 | AGAATTGCCTTGATTTCCCGGTTAGAAACTTCGACCTGCCCACTTGTTTGGGGATGATACGGTGTCGCAA |
| Lo2-147 | g13 | IV | 1 | 1 | AGAATTGCCTTGATTTCCCGGTTAGAAACTTCGACCTGCCCACTTGTTTGGGGATGATACGGTGTCGCAA |
| Lo2-147 | g15 | IV | 1 | 1 | AGAATTGCCTTGATTTCCCGGTTAGAAACTTCGACCTGCCCACTTGTTTGGGGATGATACGGTGTCGCAA |
| Lo2-147 | g15 | II | 1 | 0 | AGAATTGCCTTGATTTCCCGGTTAGAAACTTCGACCTGCCCACTTGTTTGGGGATGATACGGTGTCGCAA |
| Lo2-147 | g16 | IV | 1 | 1 | AGAATTGCCTTGATTTCCCGGTTAGAAACTTCGACCTGCCCACTTGTTTGGGGATGATACGGTGTCGCAA |
| Lo2-147 | g17 | IV | 1 | 1 | AGAATTGCCTTGATTTCCCGGTTAGAAACTTCGACCTGCCCACTTGTTTGGGGATGATACGGTGTCGCAA |
| Lo2-147 | g18 | IV | 1 | 1 | AGAATTGCCTTGATTTCCCGGTTAGAAACTTCGACCTGCCCACTTGTTTGGGGATGATACGGTGTCGCAA |
| Lo2-147 | g18 | III | 0 | 1 | AGAATTGCCTTGATTTCCCGGTTAGAAACTTCGACCTGCCCACTTGTTTGGGGATGATACGGTGTCGCAA |
| Lo2-147 | g19 | IV | 1 | 1 | AGAATTGCCTTGATTTCCCGGTTAGAAACTTCGACCTGCCCACTTGTTTGGGGATGATACGGTGTCGCAA |
| Lo2-147 | g20 | IV | 1 | 1 | AGAATTGCCTTGATTTCCCGGTTAGAAACTTCGACCTGCCCACTTGTTTGGGGATGATACGGTGTCGCAA |
| Lo2-147 | g20 | IV | 1 | 1 | AGAATTGCCTTGATTTCCCGGTTAGAAACTTCGACCTGCCCACTTGTTTGGGGATGATACGGTGTCGCAA |
| Lo2-147 | g21 | IV | 1 | 1 | AGAATTGCCTTGATTTCCCGGTTAGAAACTTCGACCTGCCCACTTGTTTGGGGATGATACGGTGTCGCAA |
| Lo2-147 | g22 | II | 1 | 0 | AGAATTGCCTTGATTTCCCGGTTAGAAACTTCGACCTGCCCACTTGTTTGGGGATGATACGGTGTCGCAA |
| Lo2-147 | g23 | IV | 1 | 1 | AGAATTGCCTTGATTTCCCGGTTAGAAACTTCGACCTGCCCACTTGTTTGGGGATGATACGGTGTCGCAA |
| Lo2-147 | g24 | IV | 1 | 1 | AGAATTGCCTTGATTTCCCGGTTAGAAACTTCGACCTGCCCACTTGTTTGGGGATGATACGGTGTCGCAA |
| Lo2-147 | g25 | IV | 1 | 1 | AGAATTGCCTTGATTTCCCGGTTAGAAACTTCGACCTGCCCACTTGTTTGGGGATGATACGGTGTCGCAA |
| Lo2-147 | g70 | IV | 1 | 1 | AGAATTGCCTTGATTTCCCGGTTAGAAACTTCGACCTGCCCACTTGTTTGGGGATGATACGGTGTCGCAA |
| Lo2-147 | g94 | IV | 1 | 1 | AGAATTGCCTTGATTTCCCGGTTAGAAACTTCGACCTGCCCACTTGTTTGGGGATGATACGGTGTCGCAA |
| Lo2-147 | g99 | IV | 1 | 1 | AGAATTGCCTTGATTTCCCGGTTAGAAACTTCGACCTGCCCACTTGTTTGGGGATGATACGGTGTCGCAA |
| Lo2-147 | g113 | IV | 1 | 1 | AGAATTGCCTTGATTTCCCGGTTAGAAACTTCGACCTGCCCACTTGTTTGGGGATGATACGGTGTCGCAA |
| Lo2-265 | g2 | IV | 1 | 1 | CGAGTTATAACAATAGAGGAAGTTTCCGGTTAGTAGCGAGAAAACGACGGCGTCGAAATT |
| Lo2-265 | g2 | IV | 1 | 1 | CGAGTTATAACAATAGAGGAAGTTTCCGGTTAGTAGCGAGAAAACGACGGCGTCGAAATT |
| Lo2-265 | g2 | IV | 1 | 1 | CGAGTTATAACAATAGAGGAAGTTTCCGGTTAGTAGCGAGAAAACGACGGCGTCGAAATT |
| Lo2-265 | g20 | IV | 1 | 1 | CGAGTTATAACAATAGAGGAAGTTTCCGGTTAGTAGCGAGAAAACGACGGCGTCGAAATT |
| Lo2-265 | g25 | IV | 1 | 1 | CGAGTTATAACAATAGAGGAAGTTTCCGGTTAGTAGCGAGAAAACGACGGCGTCGAAATT |
| Lo2-265 | g94 | IV | 1 | 1 | CGAGTTATAACAATAGAGGAAGTTTCCGGTTAGTAGCGAGAAAACGACGGCGTCGAAATT |
| Lo2-265 | g99 | IV | 1 | 1 | CGAGTTATAACAATAGAGGAAGTTTCCGGTTAGTAGCGAAAAAACGACGGCGTCGAAATT |
| Lo2-265 | g113 | IV | 1 | 1 | CGAGTTATAACAATAGAGGAAGTTTCCGGTTAGTAGCGAGAAAACGACGGCGTCGAAATT |
| ^a^ The number is based on mixed scoring (see text) | | | | | |
| ^b^ "1" represents "uncut" (methylation or sequence difference) | | | | | |
