# Supplementary material for: Genet-specific DNA methylation probabilities detected in a spatial epigenetic analysis of a clonal plant population
Source: PLoS One. 2017 May 22;12(5):e0178145. doi: 10.1371/journal.pone.0178145 (PMC5439711; doi:10.1371/journal.pone.0178145)
Supplement: S4 Table — (DOCX) [file pone.0178145.s004.docx]

| **S4a Table. Summary of posterior distributions in the hierarchical Bayesian model for n-subepiloci of mixed scoring analysis.** | | | |
| --- | --- | --- | --- |
| Parameter | Median | 95% interval (lower, upper limits) | Deviation |
| Mean.locus | -5.59 | (-7.53, -3.73) | - |
| Mean.cover | 0.07 | (-0.17, 0.33) |  |
| Cover [Lo1-042] | 0.05 | (-0.44, 0.53) |  |
| Cover [Lo1-080] | 0.12 | (-0.41, 0.77) |  |
| Cover [Lo1-123] | 0.26 | (-0.25, 0.98) |  |
| Cover [Lo1-193] | 0.01 | (-0.68, 0.59) |  |
| Cover [Lo1-203] | 0.07 | (-0.67, 0.81) |  |
| Cover [Lo1-225] | 0.14 | (-0.52, 1.00) |  |
| Cover [Lo2-147] | -0.05 | (-0.82, 0.50) |  |
| Cover [Lo2-170] | 0.11 | (-0.25, 0.49) |  |
| Cover [Lo2-181] | 0.11 | (-0.55, 0.88) |  |
| Cover [Lo2-184] | 0.18 | (-0.36, 0.88) |  |
| Cover [Lo2-265] | 0.08 | (-0.38, 0.56) |  |
| Cover [Lo2-292] | 0.4 | (0.00, 0.89) | + |
| Cover [Lo3-082] | 0.08 | (-0.31, 0.48) |  |
| Cover [Lo3-096] | -0.2 | (-0.63, 0.16) |  |
| Cover [Lo3-100] | 0.16 | (-0.39, 0.87) |  |
| Cover [Lo3-165] | 0.23 | (-0.13, 0.66) |  |
| Cover [Lo3-257] | -0.17 | (-1.10, 0.38) |  |
| Cover [Lo3-300] | -0.19 | (-0.80, 0.23) |  |
| Cover [Lo3-325] | 0.06 | (-0.63, 0.84) |  |
| Cover [Lo3-343] | -0.16 | (-0.54, 0.19) |  |
| Cover [Lo4-075] | 0.06 | (-0.62, 0.67) |  |
| Cover [Lo4-091] | 0.17 | (-0.37, 1.09) |  |
| Cover [Lo4-147] | 0.07 | (-0.70, 0.83) |  |
| Cover [Lo4-235] | 0.15 | (-0.46, 0.96) |  |
| Locus [Lo1-042] | -5.22 | (-6.91, -3.76) | - |
| Locus [Lo1-080] | -5.3 | (-6.98, -3.84) | - |
| Locus [Lo1-123] | -6.48 | (-8.67, -4.71) | - |
| Locus [Lo1-193] | -6.75 | (-8.98, -4.93) | - |
| Locus [Lo1-203] | -10.59 | (-17.07, -6.91) | - |
| Locus [Lo1-225] | -8.43 | (-11.88, -6.11) | - |
| Locus [Lo2-147] | -6.81 | (-9.01, -4.89) | - |
| **S4a Table.** (Continued) | | | |
| Parameter | Median | 95% interval (lower, upper limits) | Deviation |
| Locus [Lo2-181] | -7.36 | (-10.11, -5.40) | - |
| Locus [Lo2-184] | -6.91 | (-9.26, -5.05) | - |
| Locus [Lo2-265] | -4.74 | (-6.18, -3.33) | - |
| Locus [Lo2-292] | -2.35 | (-3.63, -1.13) | - |
| Locus [Lo3-082] | -2.82 | (-4.08, -1.67) | - |
| Locus [Lo3-096] | 1.49 | (0.38, 2.62) | + |
| Locus [Lo3-100] | -6.84 | (-9.12, -4.96) | - |
| Locus [Lo3-165] | -3.23 | (-4.58, -2.01) | - |
| Locus [Lo3-257] | -6.5 | (-8.61, -4.76) | - |
| Locus [Lo3-300] | 3.71 | (2.43, 5.06) | + |
| Locus [Lo3-325] | -10.55 | (-16.53, -7.02) | - |
| Locus [Lo3-343] | -0.31 | (-1.42, 0.77) |  |
| Locus [Lo4-075] | -7.47 | (-10.00, -5.46) | - |
| Locus [Lo4-091] | -7.51 | (-10.05, -5.46) | - |
| Locus [Lo4-147] | -10.57 | (-16.77, -7.09) | - |
| Locus [Lo4-235] | -8.65 | (-12.04, -6.01) | - |
| Sigma [Locus] | 4.02 | (2.92, 6.07) | (always positive) |
| Sigma [Cover] | 0.31 | (0.05, 0.64) | (always positive) |
| Sigma [Genet] | 2.71 | (2.29, 3.23) | (always positive) |
| Sigma [Spatial heterogeneity] | 0.55 | (0.28, 0.83) | (always positive) |
|  |  |  |  |

| **S4b Table.** **Summary of posterior distributions in the hierarchical Bayesian model for m-subepiloci of mixed scoring analysis.** | | | |
| --- | --- | --- | --- |
| Parameter | Median | 95% interval (lower, upper limits) | Deviation |
| Mean.locus | 1.92 | (0.53, 3.39) | + |
| Mean.cover | -0.04 | (-0.20, 0.13) |  |
| Cover [Lo1-042] | -0.33 | (-0.73, 0.00) |  |
| Cover [Lo1-080] | -0.02 | (-0.46, 0.44) |  |
| Cover [Lo1-123] | -0.17 | (-0.64, 0.22) |  |
| Cover [Lo1-193] | 0.03 | (-0.36, 0.45) |  |
| Cover [Lo1-203] | 0.2 | (-0.14, 0.64) |  |
| Cover [Lo1-225] | -0.11 | (-0.53, 0.28) |  |
| Cover [Lo2-147] | 0.06 | (-0.33, 0.52) |  |
| Cover [Lo2-170] | -0.06 | (-0.40, 0.28) |  |
| Cover [Lo2-181] | -0.13 | (-0.51, 0.24) |  |
| Cover [Lo2-184] | -0.34 | (-0.70, -0.01) | - |
| Cover [Lo2-265] | 0.02 | (-0.34, 0.45) |  |
| Cover [Lo2-292] | 0.06 | (-0.28, 0.44) |  |
| Cover [Lo3-082] | -0.01 | (-0.38, 0.37) |  |
| Cover [Lo3-096] | 0.21 | (-0.14, 0.63) |  |
| Cover [Lo3-100] | -0.01 | (-0.50, 0.46) |  |
| Cover [Lo3-165] | -0.3 | (-0.74, 0.05) |  |
| Cover [Lo3-257] | 0.11 | (-0.30, 0.62) |  |
| Cover [Lo3-300] | 0.23 | (-0.19, 0.77) |  |
| Cover [Lo3-325] | -0.03 | (-0.52, 0.52) |  |
| Cover [Lo3-343] | 0.05 | (-0.32, 0.45) |  |
| Cover [Lo4-075] | -0.03 | (-0.39, 0.32) |  |
| Cover [Lo4-091] | 0.08 | (-0.31, 0.54) |  |
| Cover [Lo4-147] | -0.27 | (-0.82, 0.14) |  |
| Cover [Lo4-235] | -0.18 | (-0.56, 0.15) |  |
| Locus [Lo1-042] | 1.27 | (0.08, 2.52) | + |
| Locus [Lo1-080] | 4.9 | (3.36, 6.50) | + |
| Locus [Lo1-123] | 5.13 | (3.62, 6.84) | + |
| Locus [Lo1-193] | 3.6 | (2.31, 5.03) | + |
| Locus [Lo1-203] | -2.46 | (-3.80, -1.16) | - |
| Locus [Lo1-225] | 3.17 | (1.84, 4.58) | + |
| Locus [Lo2-147] | -3.66 | (-5.26, -2.25) | - |
| **S4b Table.** (Continued) | | | |
| Parameter | Median | 95% interval (lower, upper limits) | Deviation |
| Locus [Lo2-181] | 3 | (1.78, 4.35) | + |
| Locus [Lo2-184] | 1.07 | (-0.11, 2.25) |  |
| Locus [Lo2-265] | 4.46 | (3.04, 5.96) | + |
| Locus [Lo2-292] | -2.23 | (-3.56, -1.00) | - |
| Locus [Lo3-082] | 2.66 | (1.41, 4.04) | + |
| Locus [Lo3-096] | -1.78 | (-3.11, -0.60) | - |
| Locus [Lo3-100] | 5.72 | (4.06, 7.52) | + |
| Locus [Lo3-165] | 3.19 | (1.88, 4.57) | + |
| Locus [Lo3-257] | 4.9 | (3.42, 6.52) | + |
| Locus [Lo3-300] | -4.44 | (-6.00, -3.00) | - |
| Locus [Lo3-325] | 6.49 | (4.78, 8.67) | + |
| Locus [Lo3-343] | -2.78 | (-4.11, -1.44) | - |
| Locus [Lo4-075] | 0.52 | (-0.74, 1.72) |  |
| Locus [Lo4-091] | 3.99 | (2.64, 5.40) | + |
| Locus [Lo4-147] | 5.13 | (3.58, 6.89) | + |
| Locus [Lo4-235] | 2.54 | (1.29, 3.81) | + |
| Sigma [Locus] | 3.45 | (2.55, 4.81) | (always positive) |
| Sigma [Cover] | 0.27 | (0.09, 0.45) | (always positive) |
| Sigma [Genet] | 3.05 | (2.70, 3.46) | (always positive) |
| Sigma [Spatial heterogeneity] | 0.64 | (0.47, 0.82) | (always positive) |

| **S4c Table. Summary of posterior distributions in the hierarchical Bayesian model for h-subepiloci of mixed scoring analysis.** | | | |
| --- | --- | --- | --- |
| Parameter | Median | 95% interval (lower, upper limits) | Deviation |
| Mean.locus | -6.24 | (-7.42, -5.34) | - |
| Mean.cover | 0.17 | (-0.17, 0.50) |  |
| Cover [Lo1-042] | 0.19 | (-0.30, 0.82) |  |
| Cover [Lo1-080] | 0.16 | (-0.45, 0.80) |  |
| Cover [Lo1-123] | 0.19 | (-0.34, 0.83) |  |
| Cover [Lo1-193] | 0.16 | (-0.50, 0.77) |  |
| Cover [Lo1-203] | 0.21 | (-0.32, 0.96) |  |
| Cover [Lo1-225] | 0.17 | (-0.39, 0.83) |  |
| Cover [Lo2-147] | 0.15 | (-0.34, 0.63) |  |
| Cover [Lo2-170] | 0.16 | (-0.46, 0.77) |  |
| Cover [Lo2-181] | 0.08 | (-0.83, 0.53) |  |
| Cover [Lo2-184] | 0.22 | (-0.25, 0.93) |  |
| Cover [Lo2-265] | 0.13 | (-0.67, 0.64) |  |
| Cover [Lo2-292] | 0.13 | (-0.41, 0.59) |  |
| Cover [Lo3-082] | 0.16 | (-0.46, 0.77) |  |
| Cover [Lo3-096] | 0.13 | (-0.49, 0.60) |  |
| Cover [Lo3-100] | 0.16 | (-0.50, 0.82) |  |
| Cover [Lo3-165] | 0.21 | (-0.25, 0.99) |  |
| Cover [Lo3-257] | 0.13 | (-0.54, 0.65) |  |
| Cover [Lo3-300] | 0.15 | (-0.43, 0.71) |  |
| Cover [Lo3-325] | 0.16 | (-0.43, 0.79) |  |
| Cover [Lo3-343] | 0.17 | (-0.22, 0.59) |  |
| Cover [Lo4-075] | 0.18 | (-0.40, 0.80) |  |
| Cover [Lo4-091] | 0.2 | (-0.29, 0.87) |  |
| Cover [Lo4-147] | 0.22 | (-0.27, 1.00) |  |
| Cover [Lo4-235] | 0.16 | (-0.51, 0.80) |  |
| Locus [Lo1-042] | -5.87 | (-7.66, -4.54) | - |
| Locus [Lo1-080] | -7.16 | (-9.97, -5.38) | - |
| Locus [Lo1-123] | -5.91 | (-7.76, -4.55) | - |
| Locus [Lo1-193] | -7.12 | (-9.89, -5.36) | - |
| Locus [Lo1-203] | -6.38 | (-8.47, -4.82) | - |
| Locus [Lo1-225] | -6.5 | (-8.73, -4.90) | - |
| Locus [Lo2-147] | -5.32 | (-7.13, -3.99) | - |
| **S4c Table.** (Continued) | | | |
| Parameter | Median | 95% interval (lower, upper limits) | Deviation |
| Locus [Lo2-181] | -6 | (-7.95, -4.57) | - |
| Locus [Lo2-184] | -5.94 | (-7.87, -4.56) | - |
| Locus [Lo2-265] | -6.51 | (-8.81, -4.92) | - |
| Locus [Lo2-292] | -4.63 | (-6.02, -3.55) | - |
| Locus [Lo3-082] | -7.17 | (-10.10, -5.39) | - |
| Locus [Lo3-096] | -5.39 | (-7.01, -4.13) | - |
| Locus [Lo3-100] | -7.17 | (-10.01, -5.30) | - |
| Locus [Lo3-165] | -6.45 | (-8.62, -4.93) | - |
| Locus [Lo3-257] | -6 | (-7.94, -4.59) | - |
| Locus [Lo3-300] | -6.39 | (-8.43, -4.80) | - |
| Locus [Lo3-325] | -7.14 | (-9.86, -5.36) | - |
| Locus [Lo3-343] | -3.32 | (-4.56, -2.52) | - |
| Locus [Lo4-075] | -6.49 | (-8.63, -4.92) | - |
| Locus [Lo4-091] | -5.87 | (-7.64, -4.41) | - |
| Locus [Lo4-147] | -5.96 | (-7.83, -4.52) | - |
| Locus [Lo4-235] | -7.18 | (-9.97, -5.30) | - |
| Sigma [Locus] | 1.26 | (0.76, 2.19) | (always positive) |
| Sigma [Cover] | 0.17 | (0.01, 0.64) | (always positive) |
| Sigma [Genet] | 1.05 | (0.12, 1.80) | (always positive) |
| Sigma [Spatial heterogeneity] | 0.33 | (0.03, 0.94) | (always positive) |
