# Supplementary figures and images for: Genet-specific DNA methylation probabilities detected in a spatial epigenetic analysis of a clonal plant population
Source: PLoS One. 2017 May 22;12(5):e0178145. doi: 10.1371/journal.pone.0178145 (PMC5439711; doi:10.1371/journal.pone.0178145)

## Slide 1
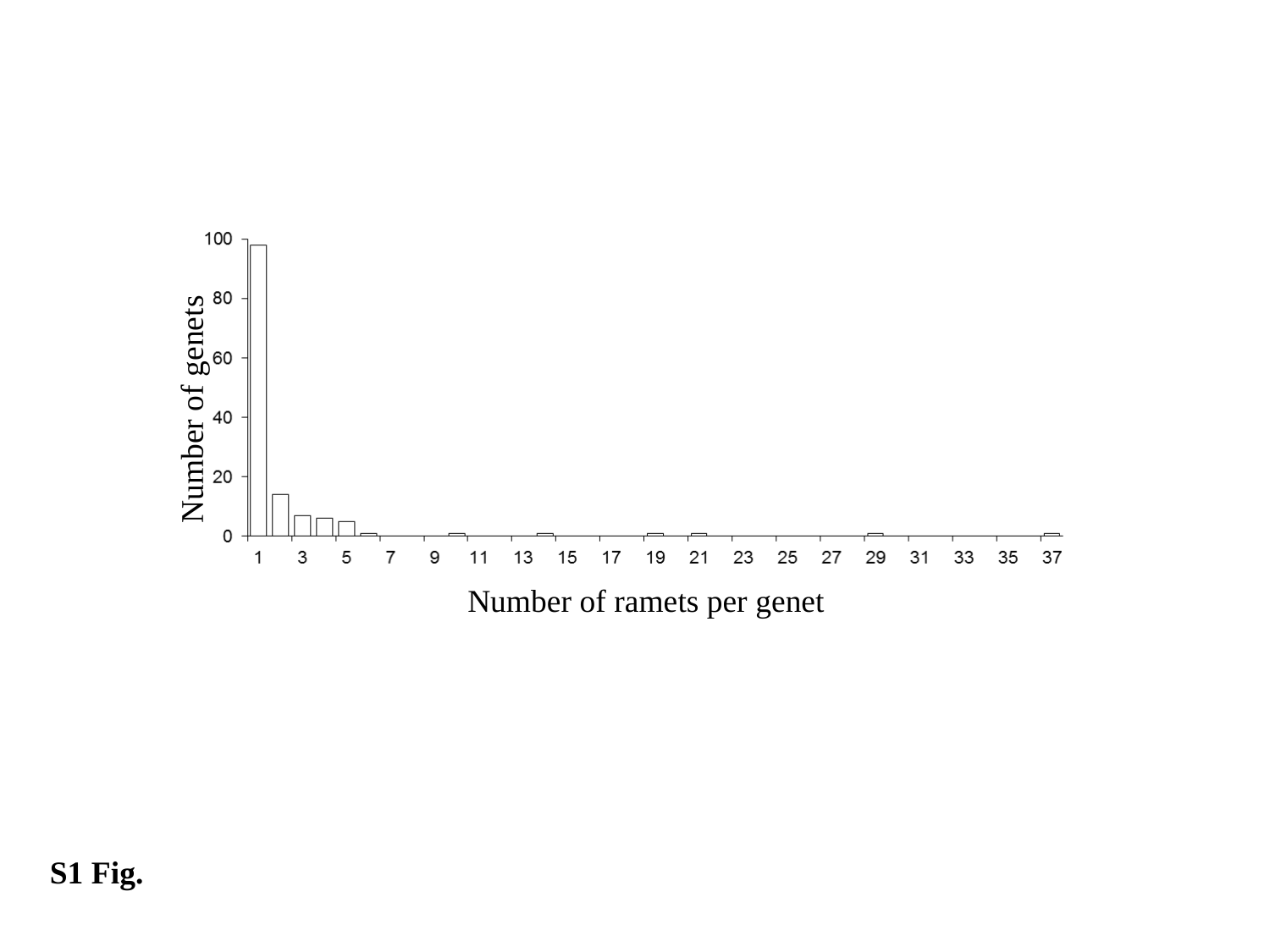

Number of genets
Number of ramets per genet
S1 Fig.

Supplement: S1 Fig — (PPT) [file pone.0178145.s005.ppt]
