# Supplementary material for: Genet-specific DNA methylation probabilities detected in a spatial epigenetic analysis of a clonal plant population
Source: PLoS One. 2017 May 22;12(5):e0178145. doi: 10.1371/journal.pone.0178145 (PMC5439711; doi:10.1371/journal.pone.0178145)

S2a Fig. Spatial distribution of genet-specific methylation statuses for n-subepiloci based on the mixed scoring analysis.

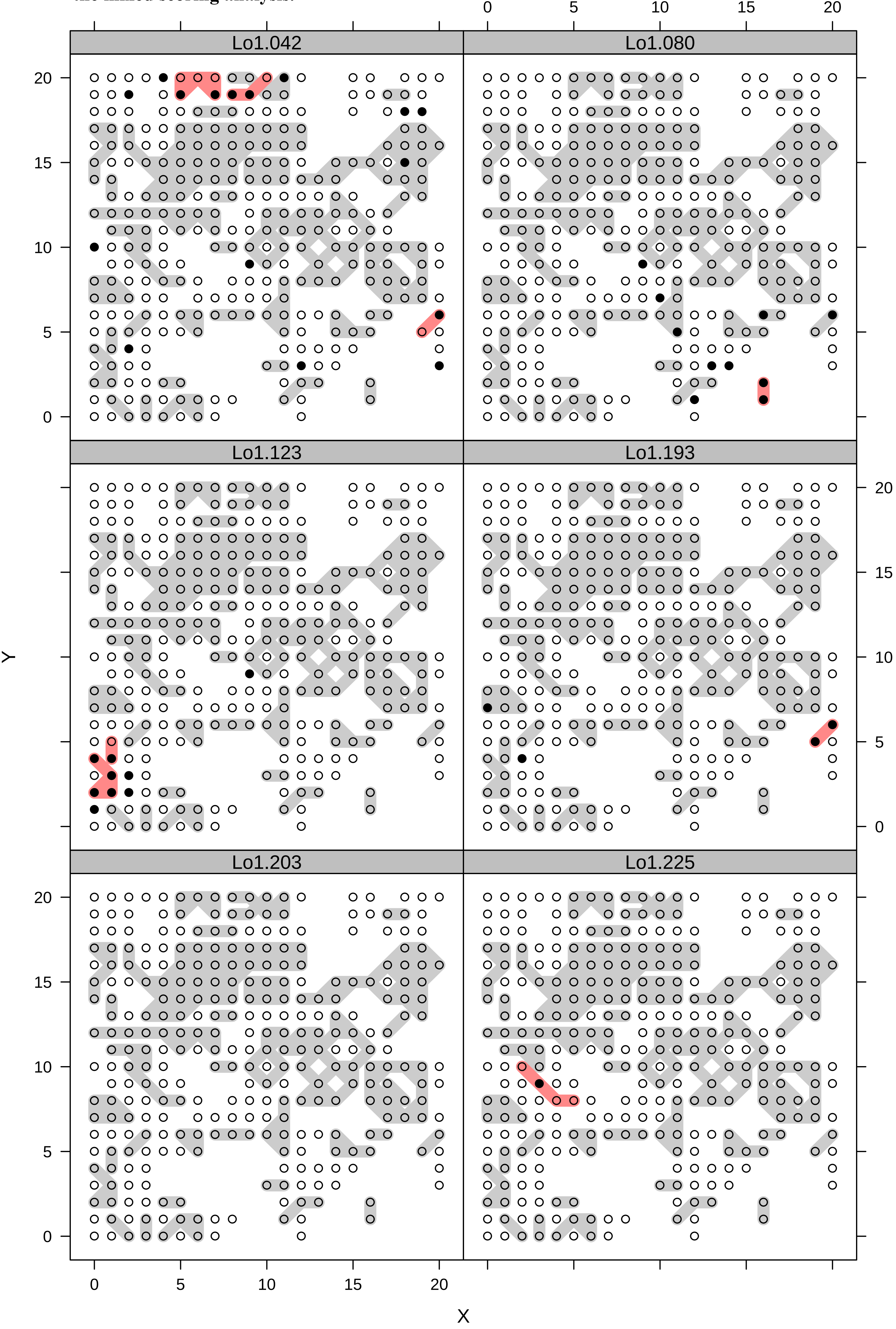

S2a Fig. (Continued)

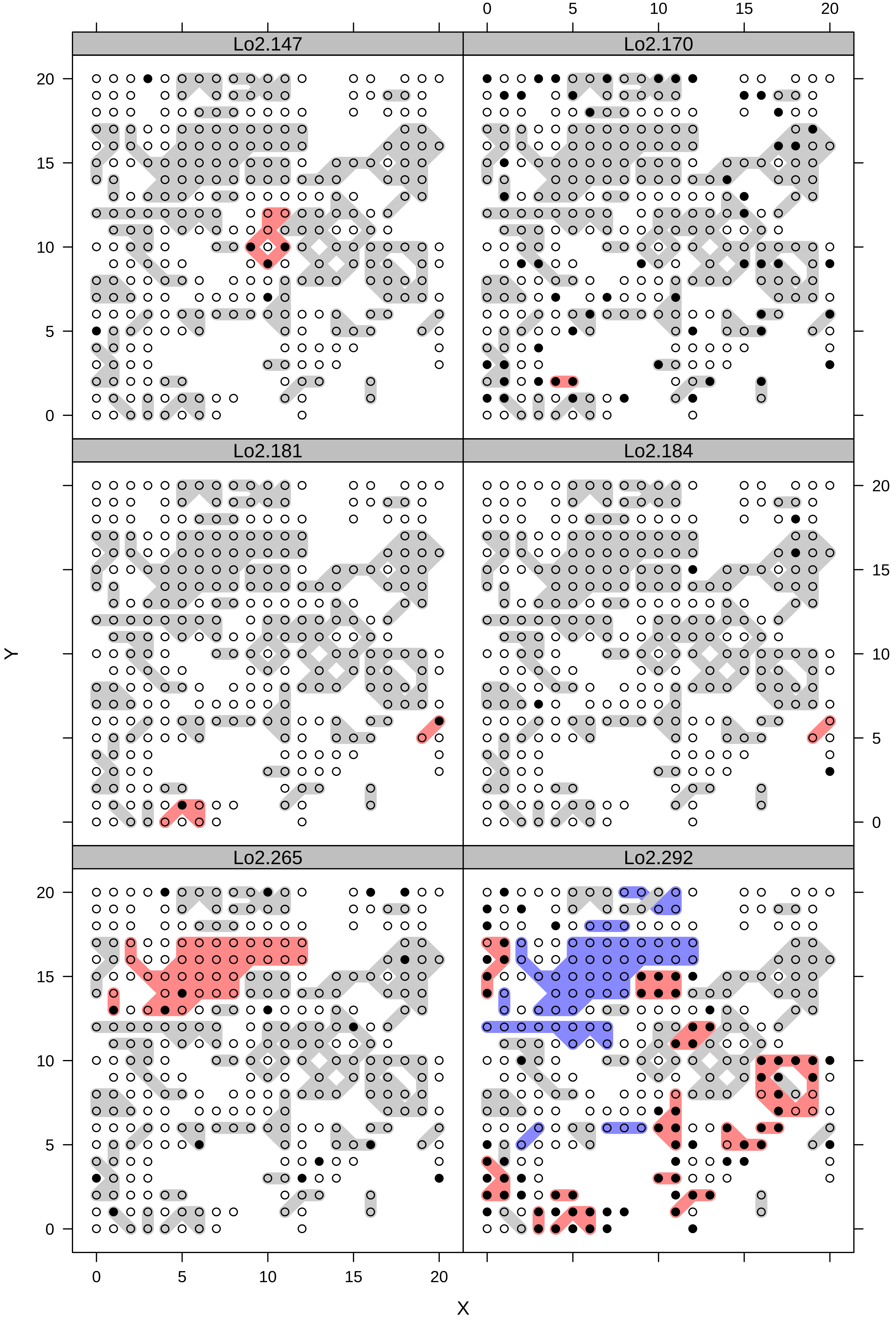

S2a Fig. (Continued)

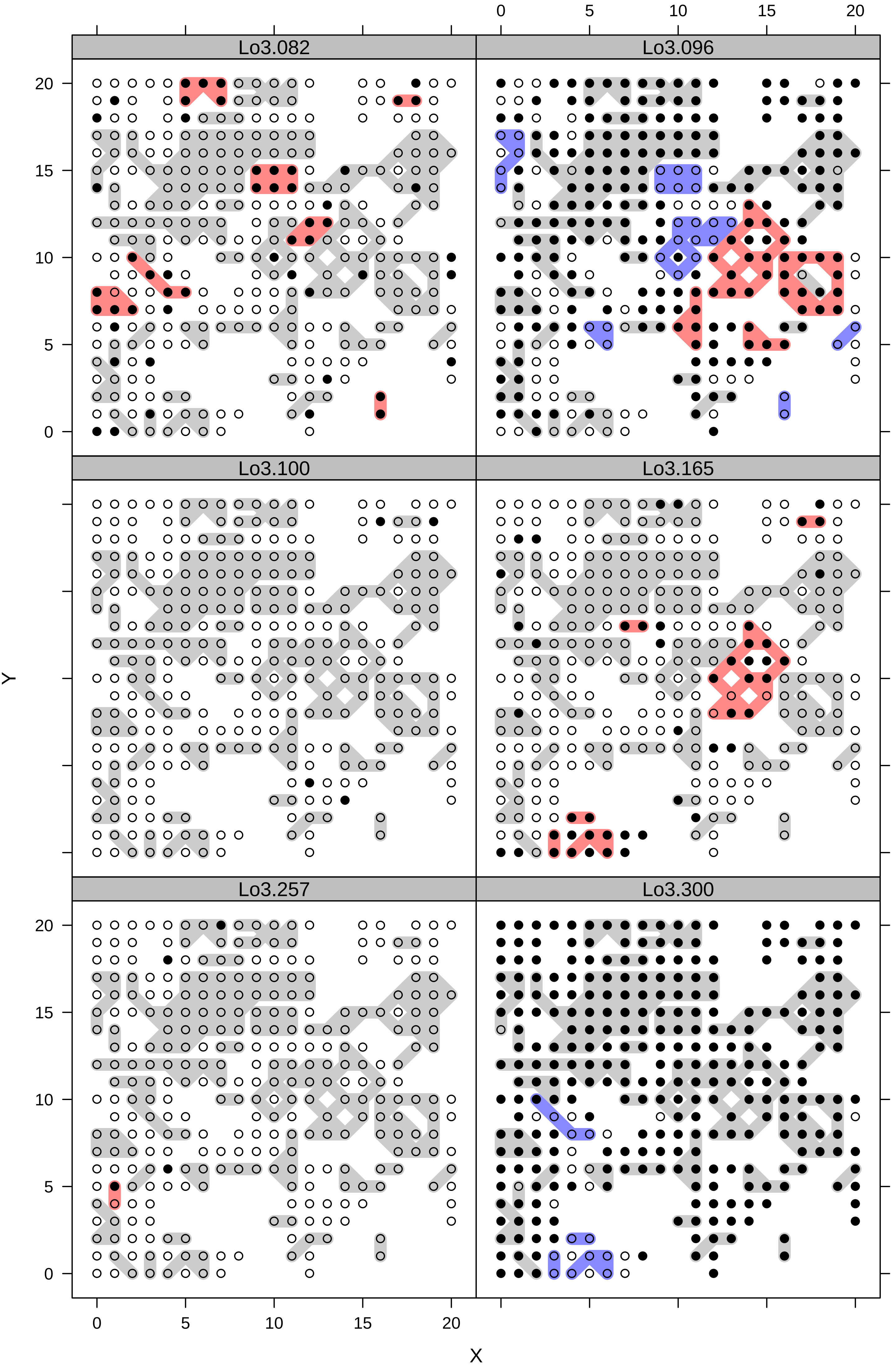

S2a Fig. (Continued)

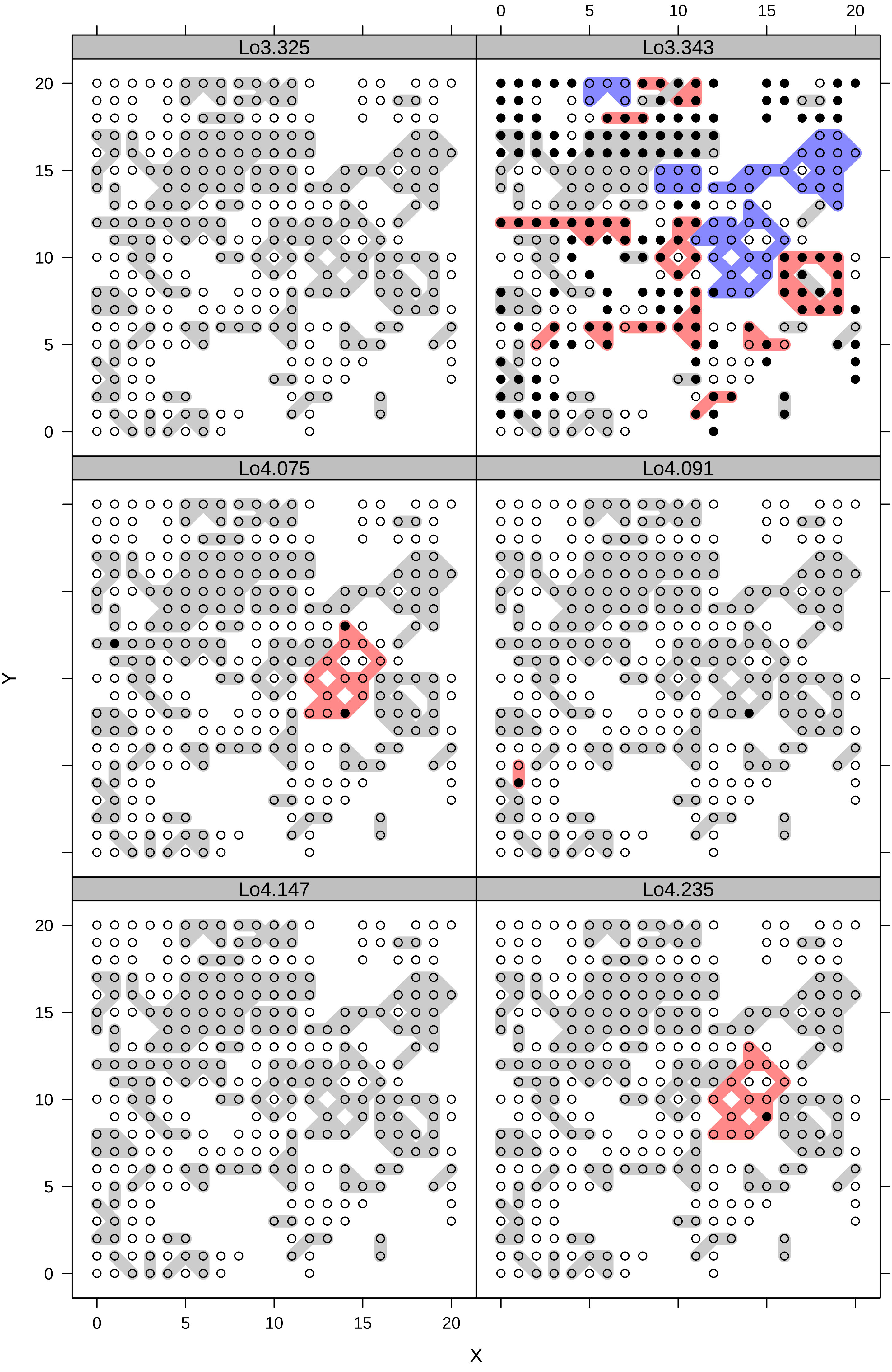

S2b Fig. Spatial distribution of genet-specific methylation statuses for m-subepiloci based on the mixed scoring analysis.

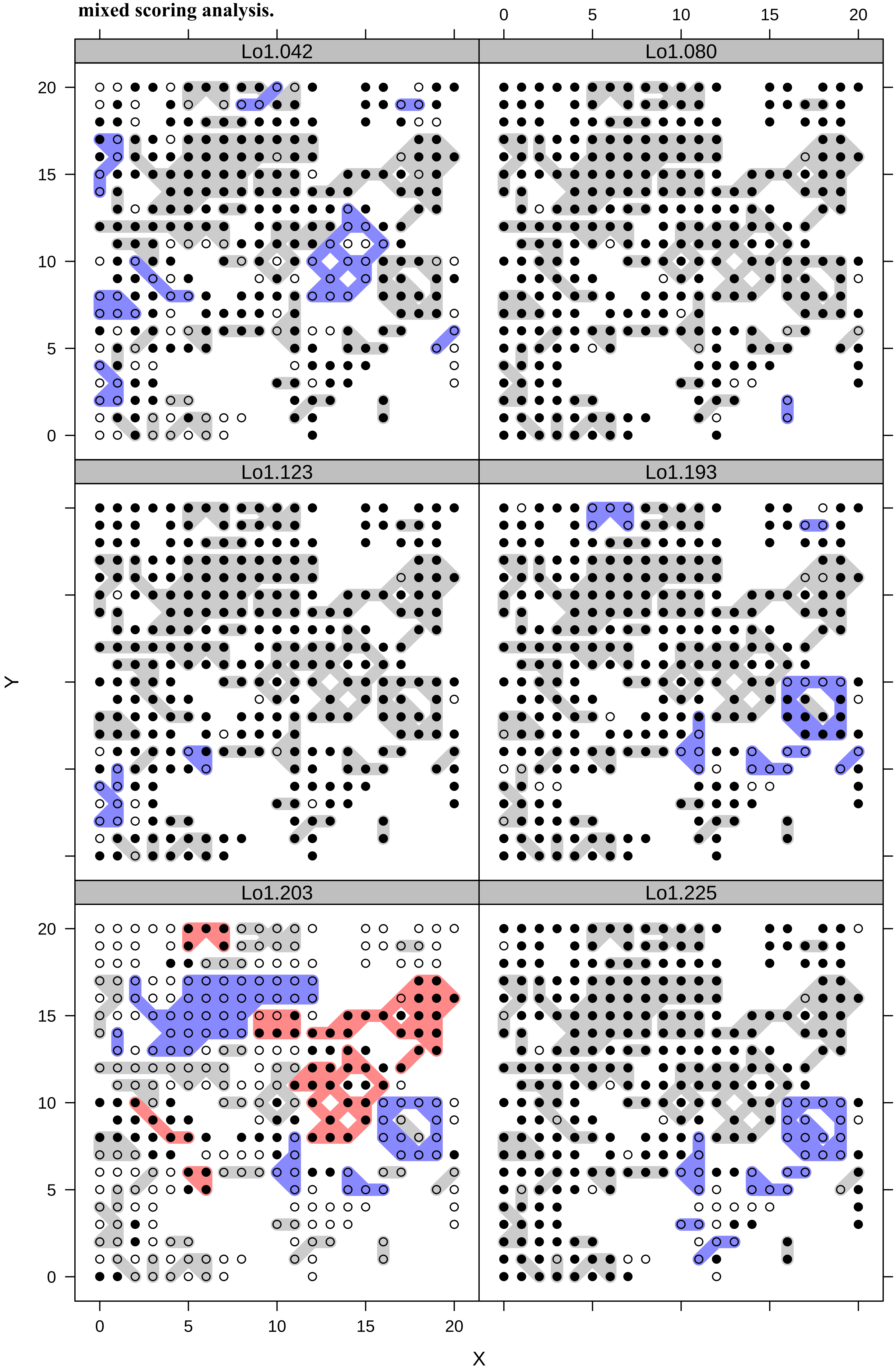

S2b Fig. (Continued)

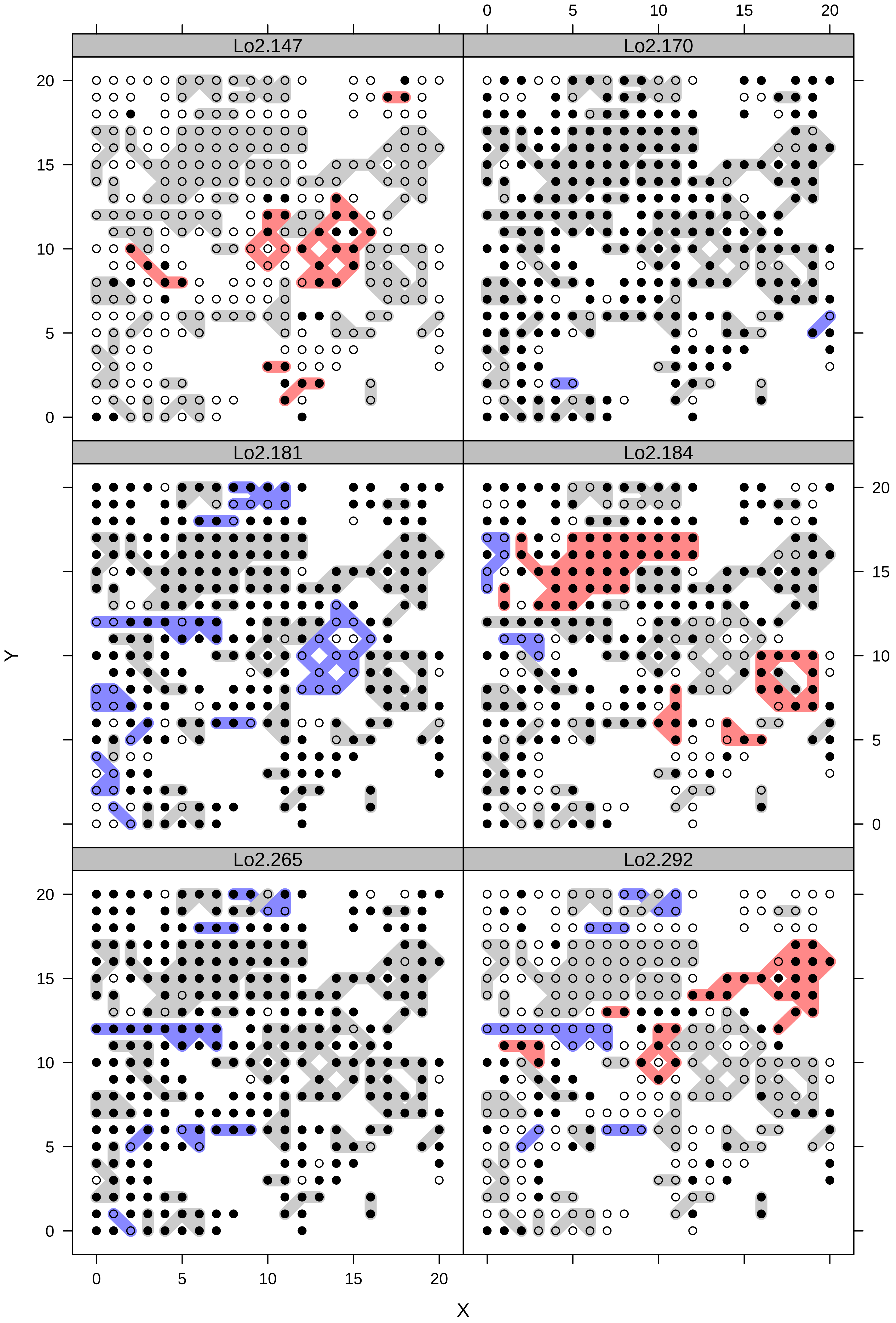

S2b Fig. (Continued)

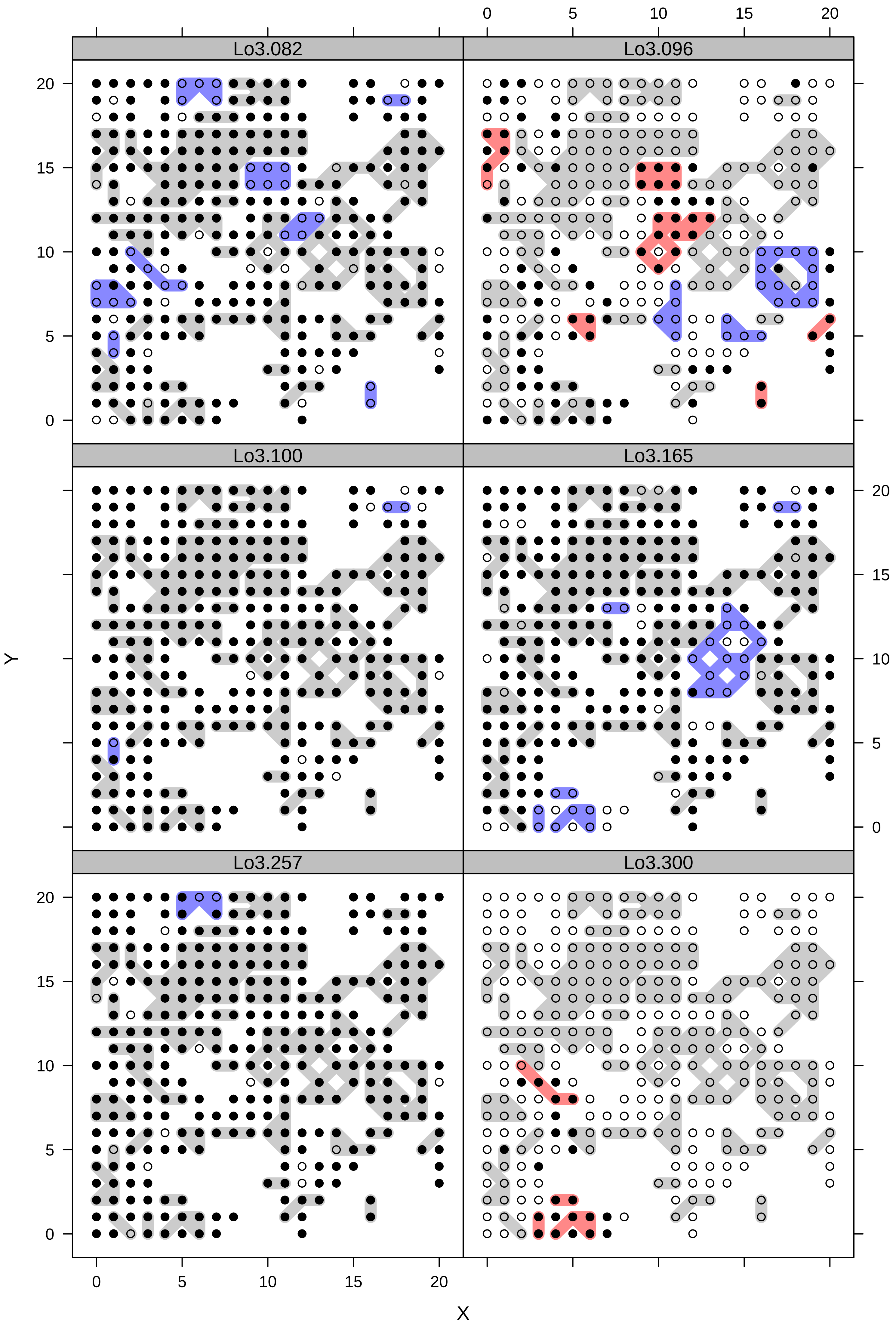

S2b Fig. (Continued)

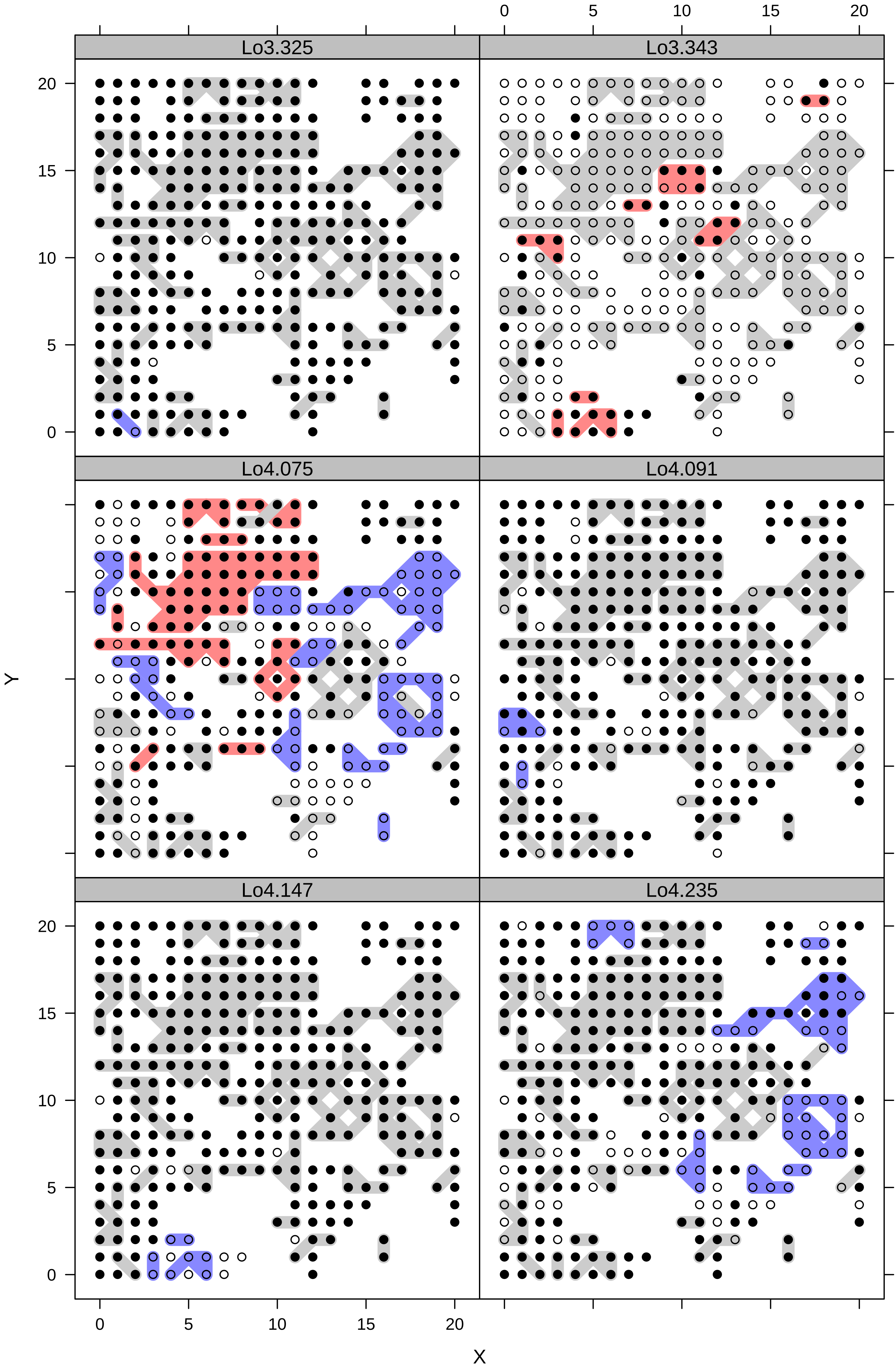

S2c Fig. Spatial distribution of genet-specific methylation statuses for h-subepiloci based on the mixed scoring analysis.

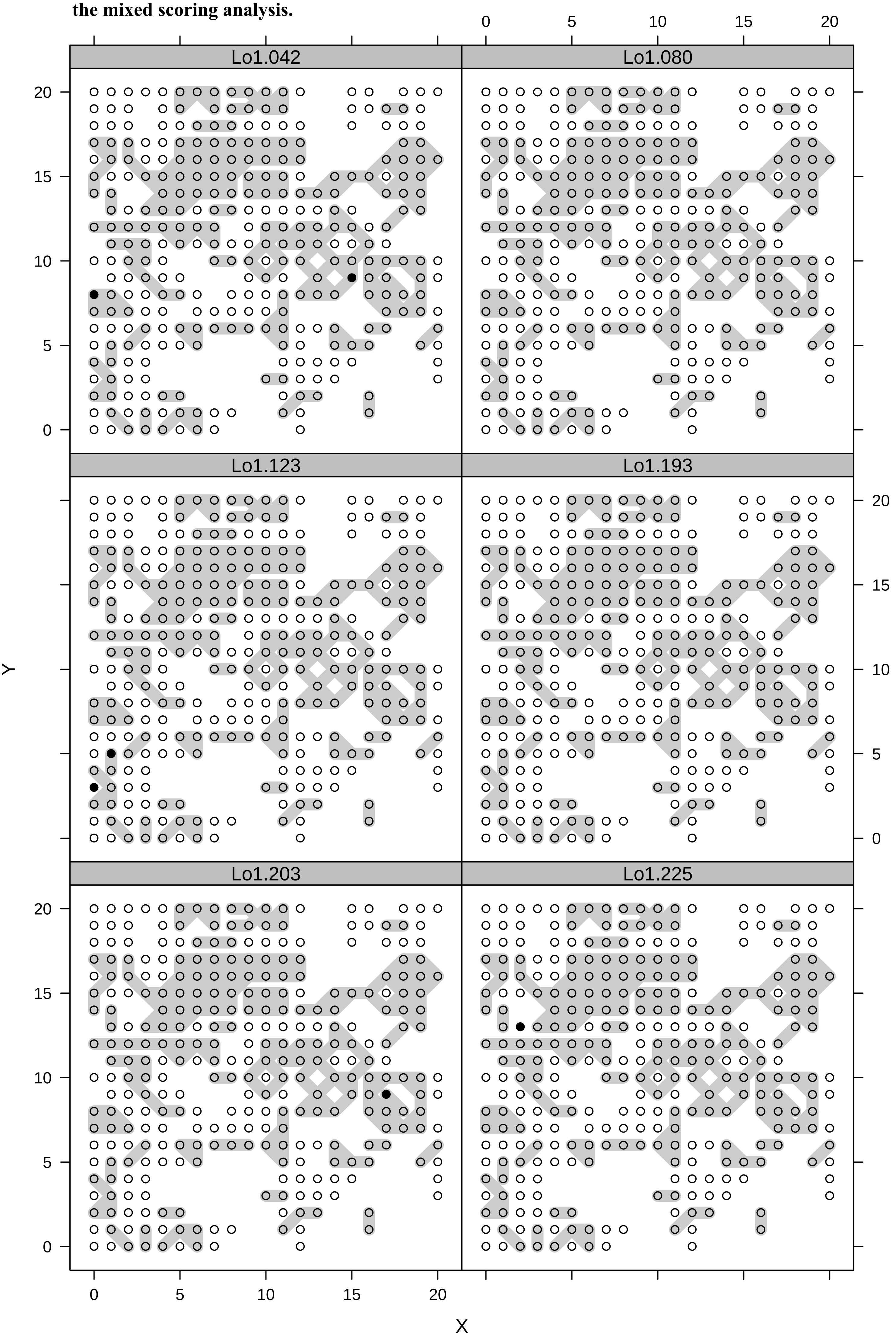

S2c Fig. (Continued)

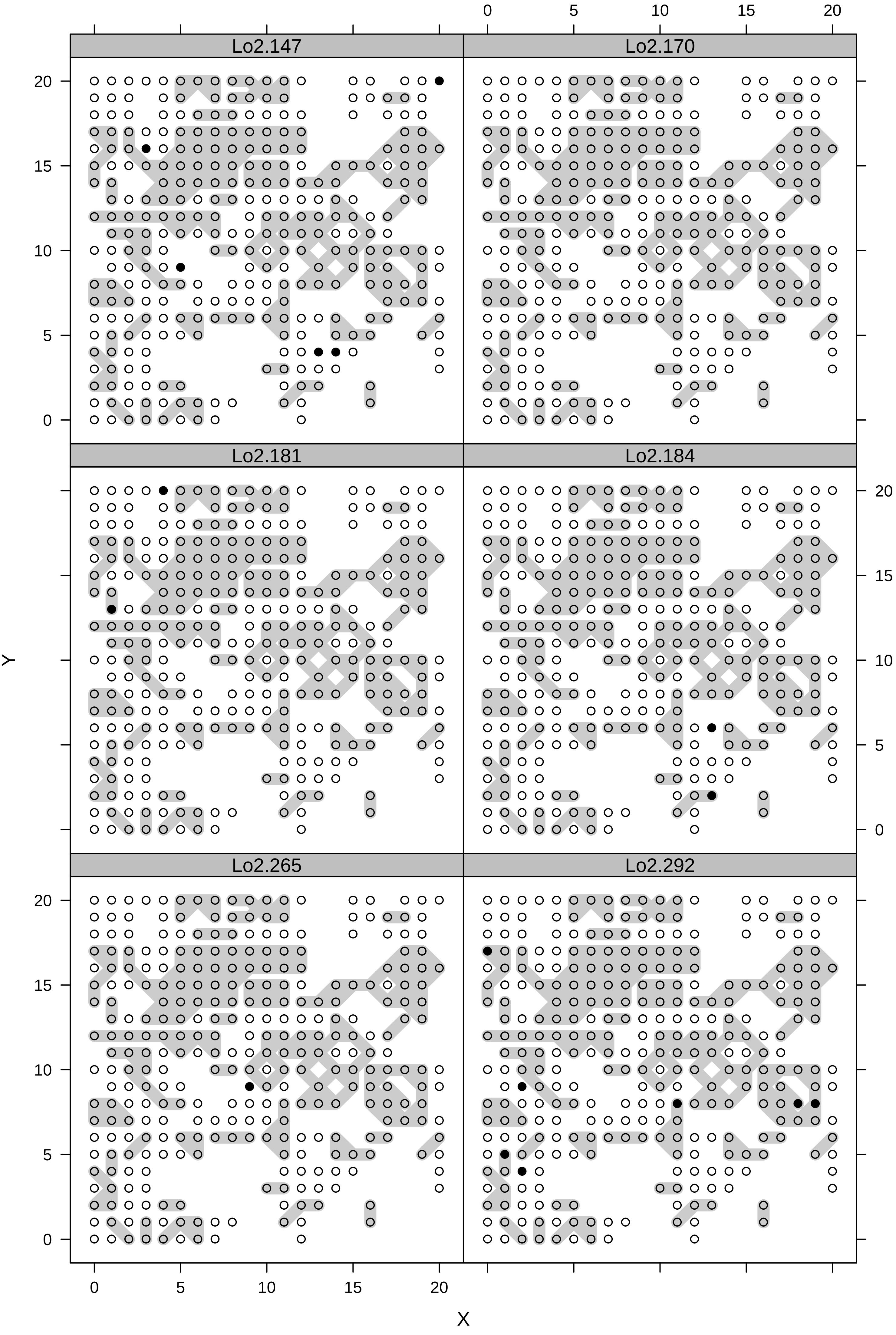

S2c Fig. (Continued)

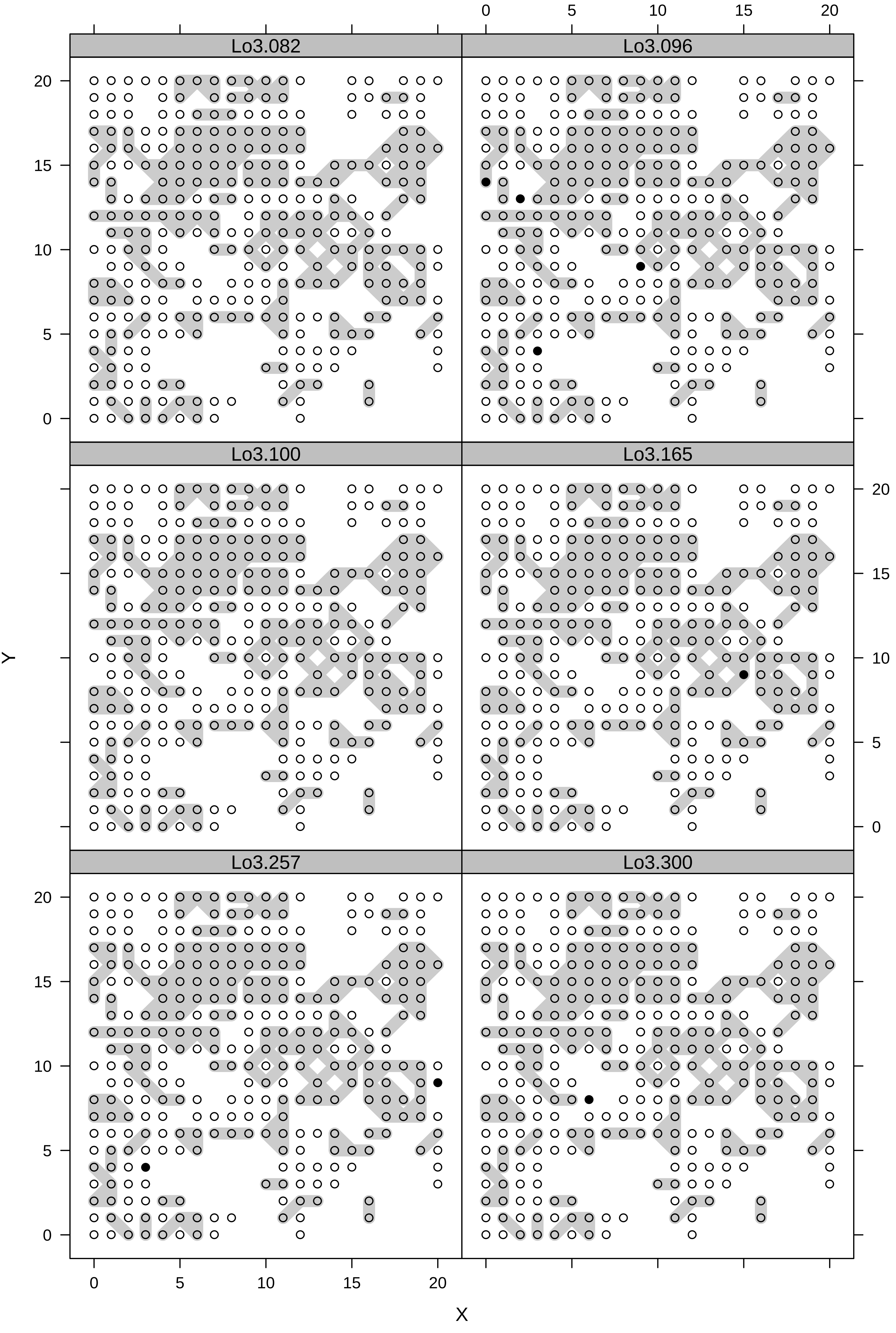

S2c Fig. (Continued)

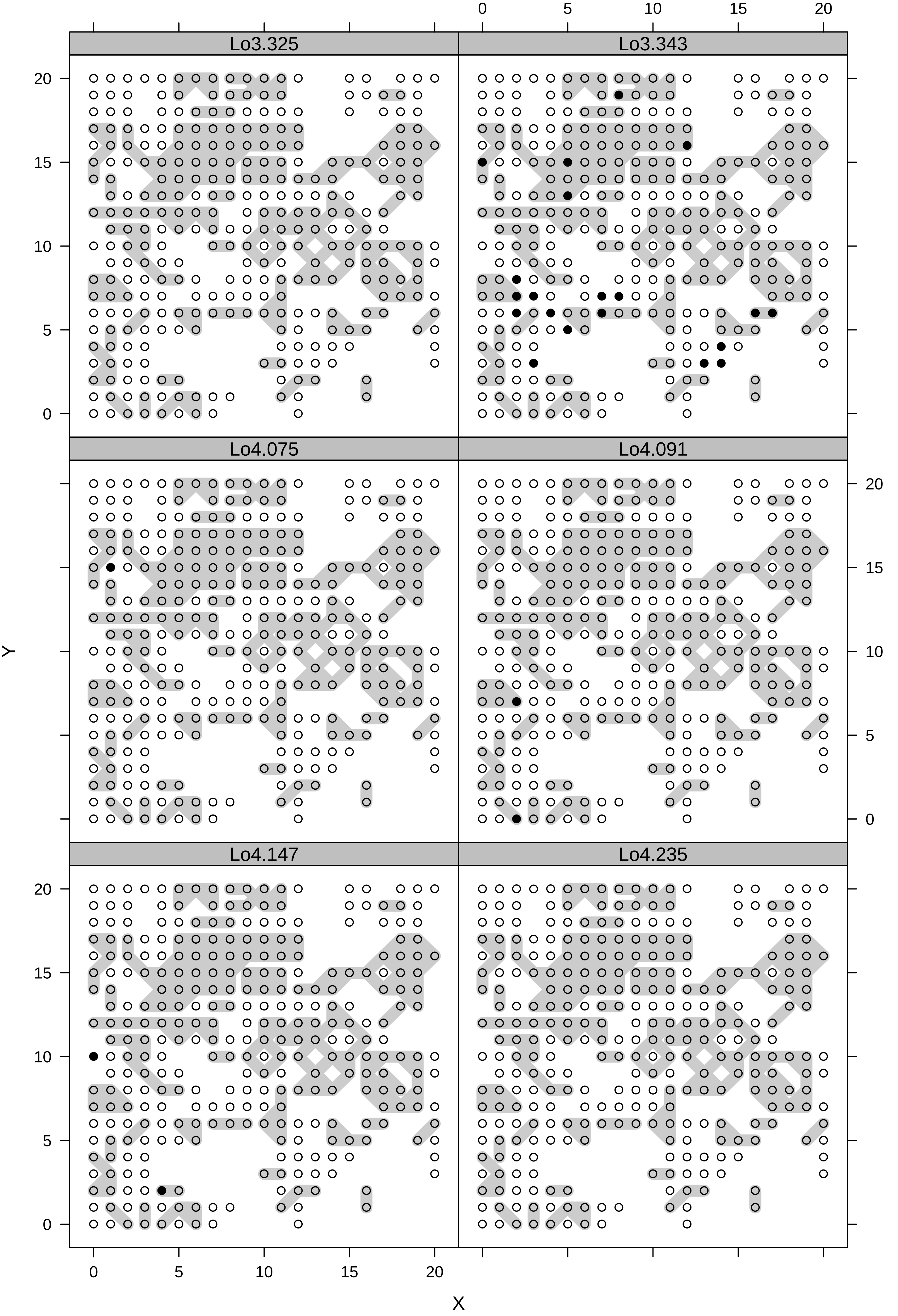

Supplement: S2 Fig — Detailed explanations are provided in Figs 1 and 6. (PDF) [file pone.0178145.s006.pdf]
