# Supplementary material for: Genet-specific DNA methylation probabilities detected in a spatial epigenetic analysis of a clonal plant population
Source: PLoS One. 2017 May 22;12(5):e0178145. doi: 10.1371/journal.pone.0178145 (PMC5439711; doi:10.1371/journal.pone.0178145)

S3a Fig. Genet-specific methylation probability for n-subepiloci based on the mixed scoring analysis.

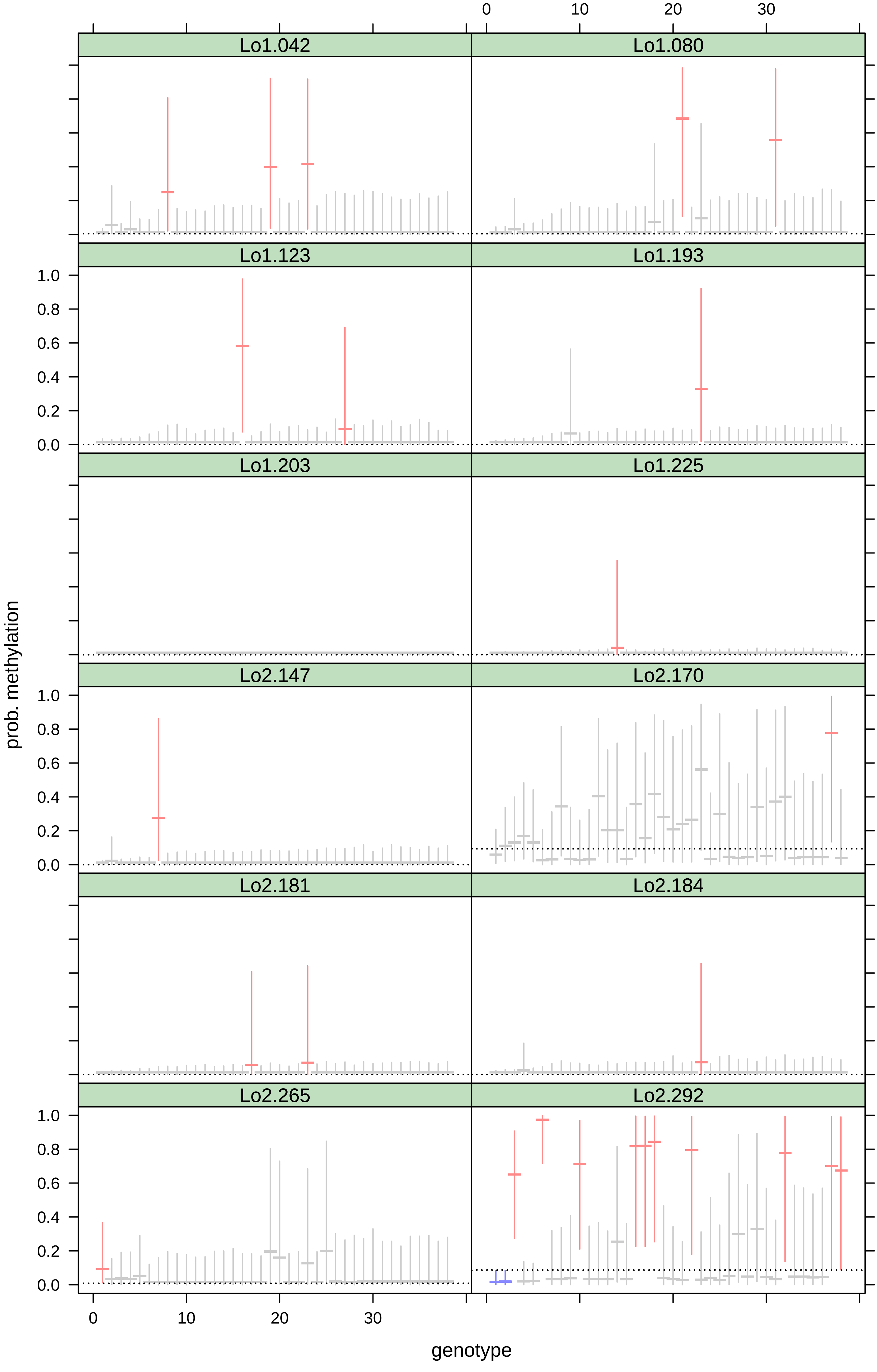

S3a Fig. (Continued)

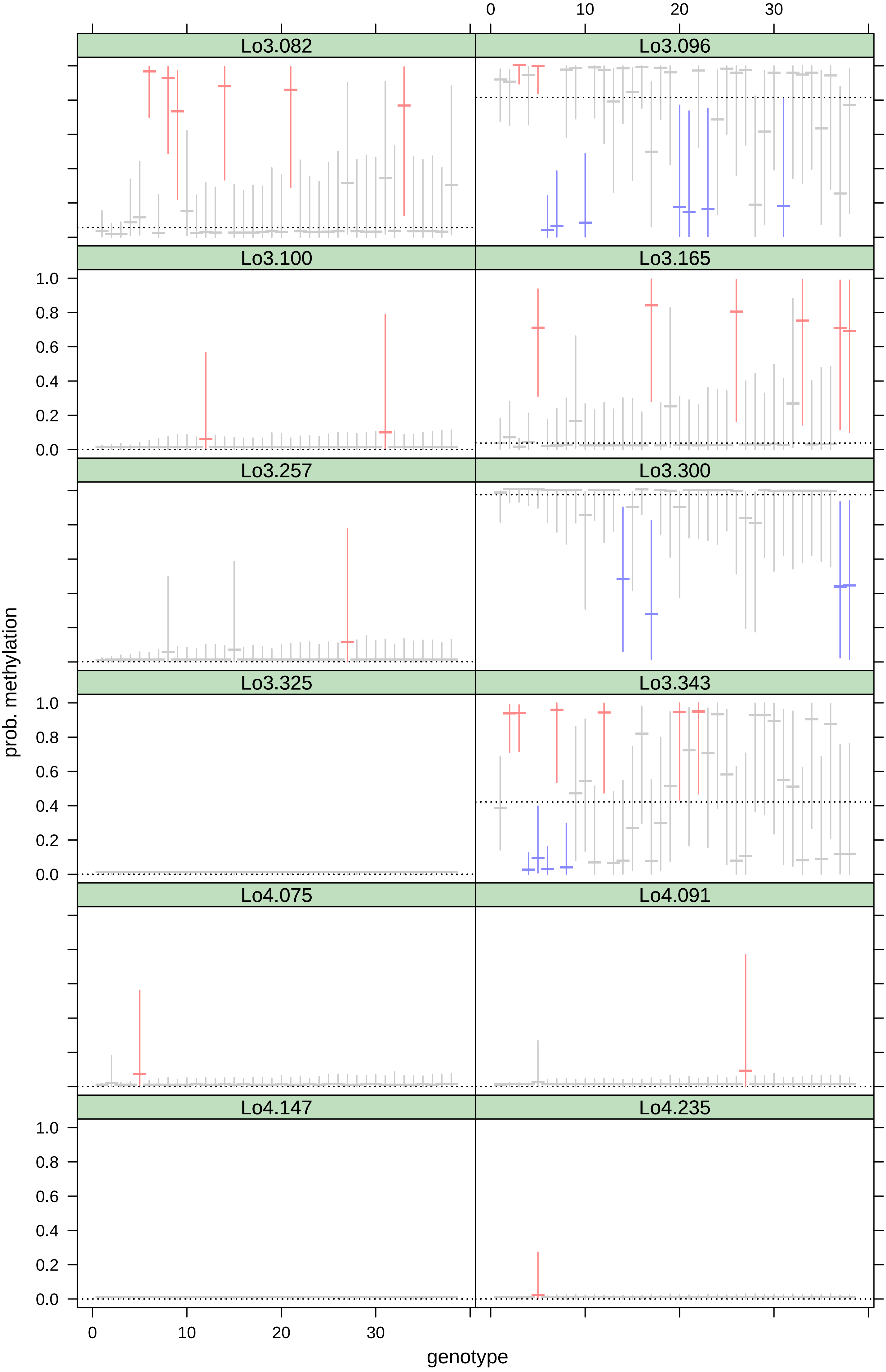

S3b Fig. Genet-specific methylation probability for m-subepiloci based on the mixed scoring analysis.

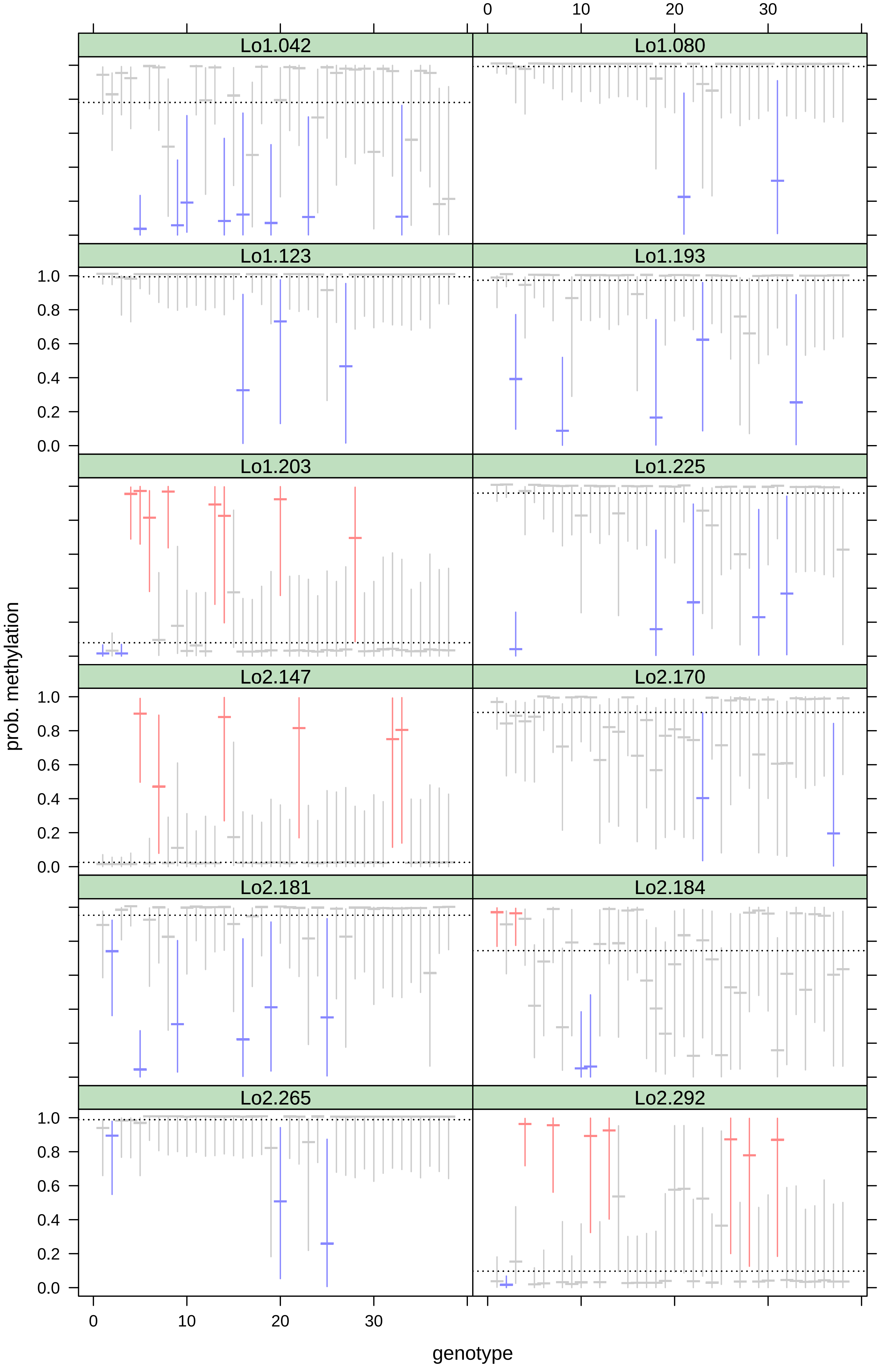

S3b Fig. (Continued)

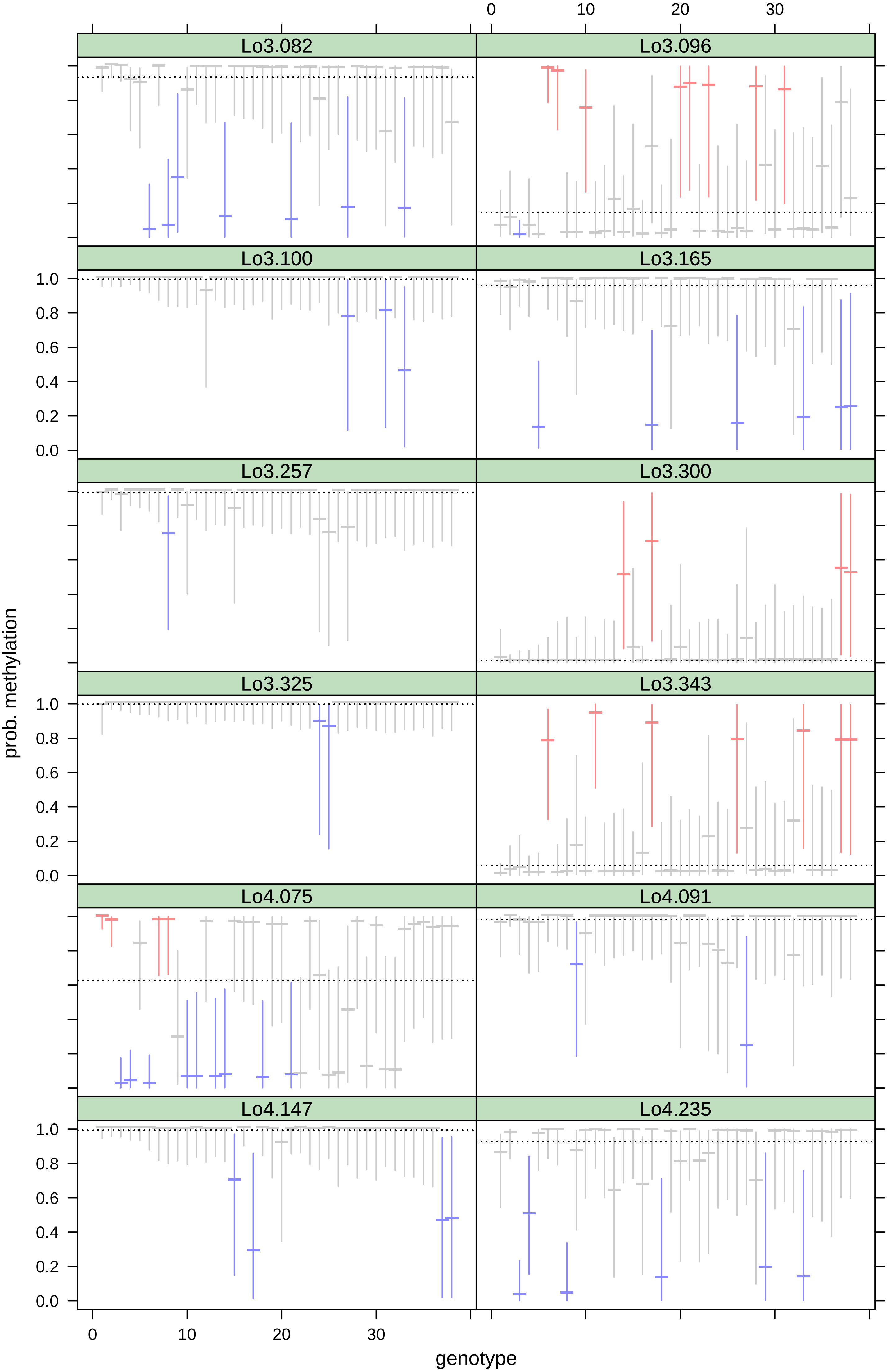

S3c Fig. Genet-specific methylation probability for h-subepiloci based on the mixed scoring analysis.

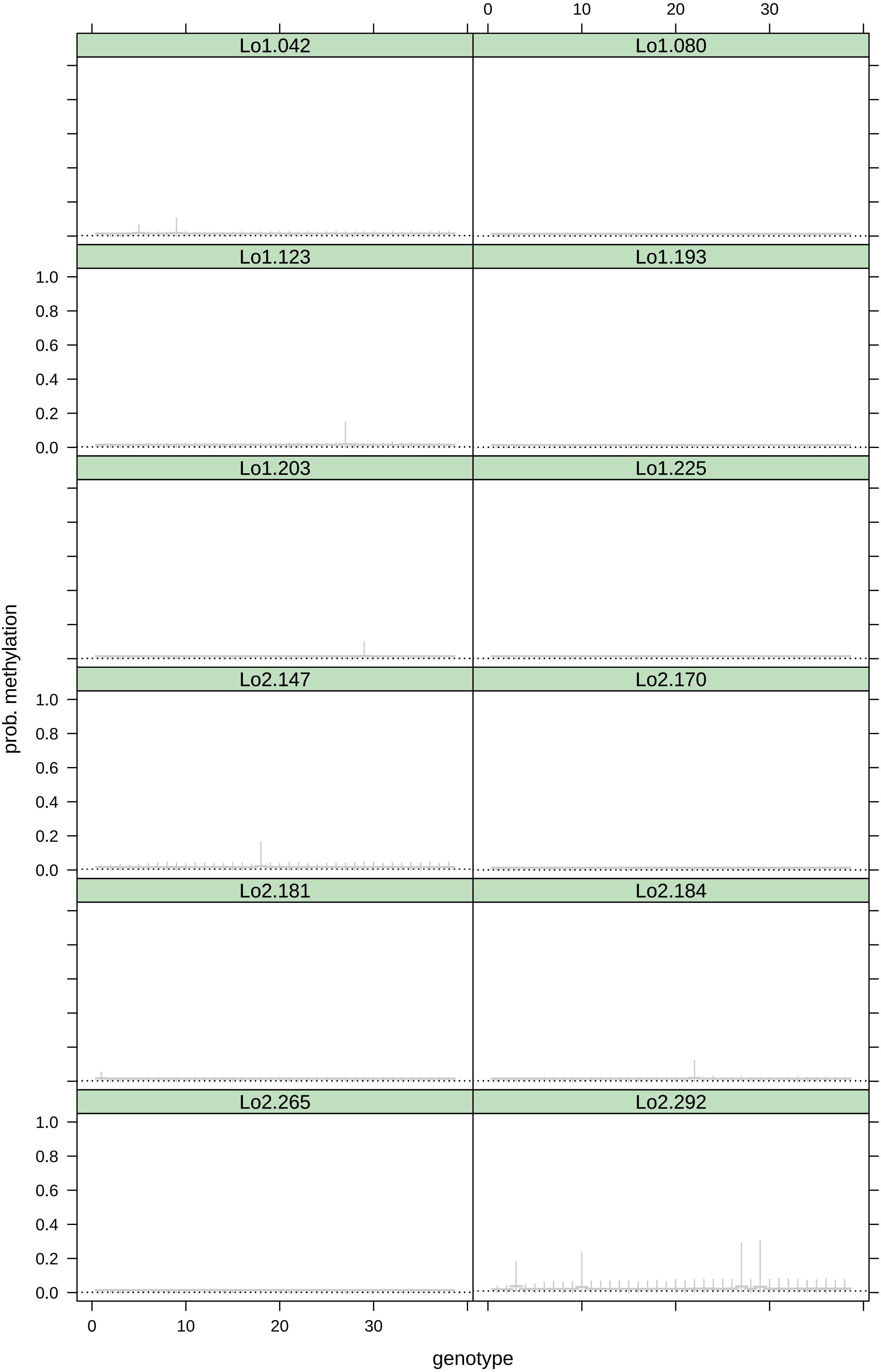

S3c Fig. (Continued)

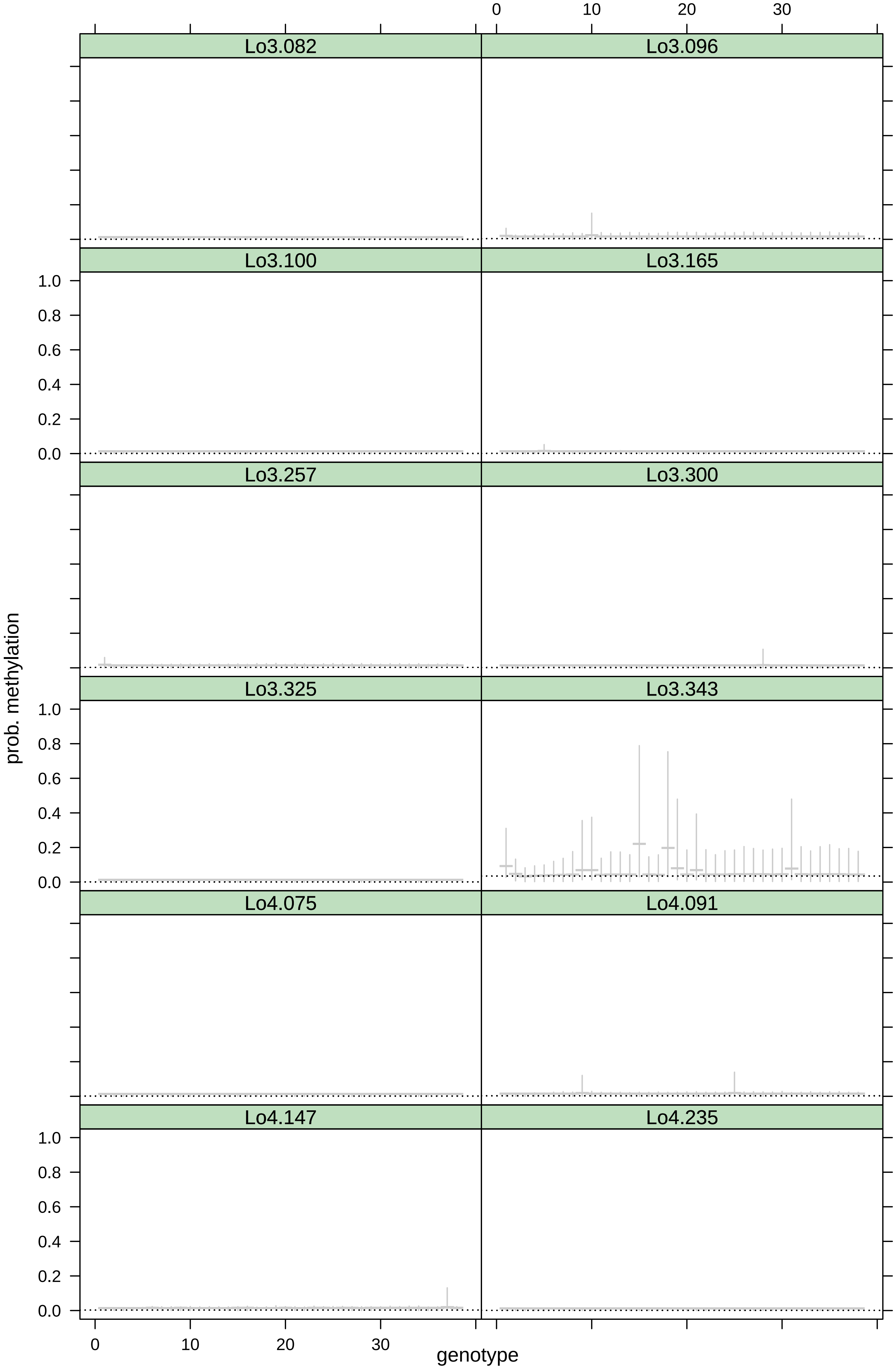

Supplement: S3 Fig — Detailed explanations are provided in Figs 1 and 6. (PDF) [file pone.0178145.s007.pdf]
