# Supplementary material for: Genet-specific DNA methylation probabilities detected in a spatial epigenetic analysis of a clonal plant population
Source: PLoS One. 2017 May 22;12(5):e0178145. doi: 10.1371/journal.pone.0178145 (PMC5439711; doi:10.1371/journal.pone.0178145)
Supplement: S1 Appendix — (DOCX) [file pone.0178145.s008.docx]

**S1 Appendix. MS-AFLP protocol.** MS-AFLP analyses were conducted using a modification of the original AFLP procedure (Reyna-Lόpez *et al*. 1997). The modified protocol involved the use of a methylation-sensitive isoschizomer, either *Hpa*II or *Msp*I, as a frequent cutter that recognizes the potential methylation site 5′-CCGG-3′. *EcoR*I (New England BioLabs, Ipswich, MA, USA) was used as the rare cutter, and the adapter and the basic primer sequences for the *EcoR*I end were the same as those used in the original protocol (Vos *et al*. 1995). A new double-stranded fragment, referred to as the *Hpa*II-*Msp*I adapter, was devised for the isoschizomer digestion by annealing the oligonucleotides 5′-*GA*TCATGAGTCCTGCT-3′ and 3′-AGTATCAGGACGA*GC*-5′ (the overhanging nucleotides are indicated in italics).

Two digestion reactions were conducted. In the first reaction, 500 ng of genomic DNA was digested with 10 U of *EcoR*I in a total volume of 50 μl containing *EcoR*I buffer at 37°C for 1.5 h. The second digestion reaction was carried out overnight with 5 U of *Hpa*II (or *Msp*I) in a final volume of 25 μl containing appropriate buffer at 37°C overnight. The digested fragments were then ligated to the adapter by adding 25 μl of the reaction mixture, containing 2.5 pmol of *EcoR*I adapter, 25 pmol of *Hpa*II-*Msp*I adapter, 5 × T4 DNA ligase buffer, 1 U of T4 DNA ligase (Invitrogen, Carlsbad, CA), 5 U of *EcoR*I, and 0.2 U of *Hpa*II (or *Msp*I) and then incubating at 20°C overnight. Five microliters of the reaction mixtures were diluted 10-fold in 0.1 × TE.

From the 57 tested primer pairs with three to ca. 50 polymorphic bands (including those with less clear patterns), we chose the four primer pairs that were most polymorphic for further analyses. Preamplification was conducted using 2.0 μl of the ligation product with three pre-selected primer pairs (E-A/HM-C, E-C/HM-T, E-A/HM-T) in a volume of 10 μl containing 7.5 μl of Amplification Core Mix (Applied Biosystems) and 2.5 ng of each primer. The reaction entailed a 2-min hold at 72°C, followed by 27 cycles of 94°C for 20 s, 56°C for 30 s, and 72°C for 2 min, with a final extension at 60°C for 30 min. The preamplified products were then diluted 20-fold in 0.1 × TE.

Selective amplifications were conducted using four primer combinations with two or three selective bases (E-AC/HM-CG, E-CT/HM-TTA, E-AC/HM-TTA, E-AGG/HM-TG). The total volume was 10 μl, containing 1.5 μl of the preamplification product, 0.5 ng of labeled *EcoR*I primer (6-FAM or VIC), 2.5 ng of *Hpa*II-*Msp*I primer, and 7.5 μl of Amplification Core Mix. The reaction entailed an initial hold at 72°C for 2 min and 10 touchdown cycles (94°C for 30 s, 66°C for 30 s, and 72°C for 2 min), during which the annealing temperature was decreased by 1 °C each cycle, followed by 20 further cycles (94°C for 30 s, 56°C for 30 s, and 72°C for 2 min); and 60°C for 30 min. The master mix was composed of 10 μl of formamide, 0.3 μl of GeneScan500 (LIZ) size standard (Applied Biosystems), 0.8 μl of blue dextran (6-FAM), and 1.2 μl of green dextran (VIC).

Fragment separation and detection were performed using an ABI PRISM 3130 genetic analyzer. First, the *EcoR*I-*Hpa*II and *EcoR*I-*Msp*I fragments of 45–500 bp in each sample were scored automatically as “presence” or “absence” for peak heights of more than 100 and less than 50, respectively, according to the standard criteria of the Gene Mapper software protocol. We then manually evaluated absence or presence for those fragments with peak height in the range 50–100 with reference to the other bands in the same sample and the same site, by visualizing electropherograms with GENEMAPPER 3.7. Finally, we selected MS-AFLP sites conservatively based on mainly clear bands with a peak height of either more than 100 or less than 50. As a result, we used only those genomic sites in which presence and absence of bands were highly distinctive for all samples. The primer pairs we selected allowed us to score 24 polymorphic methylation sites.

**References**

Reyna-López GE, Simpson J, Ruiz-Herrera J. Differences in DNA methylation patterns are detectable during the dimorphic transition of fungi by amplification of restriction polymorphisms. Mol Gen Genet. 1997; 253, 703–710.

Vos P, Hogers R, Bleeker M, Reijans M, van de Lee T, Hornes M, *et al*. AFLP: a new technique for DNA fingerprinting. Nucleic Acids Res. 1995; 23, 4407–4414.
