# Supplementary material for: Genet-specific DNA methylation probabilities detected in a spatial epigenetic analysis of a clonal plant population
Source: PLoS One. 2017 May 22;12(5):e0178145. doi: 10.1371/journal.pone.0178145 (PMC5439711; doi:10.1371/journal.pone.0178145)
Supplement: S2 Appendix — (DOCX) [file pone.0178145.s009.docx]

**S2 Appendix. Sequencing of MS-AFLP loci.** We determined the sequences of the analyzed MS-AFLP loci through selective restriction fragment amplifications. Samples with MS-AFLP bands were subjected to electrophoresis again and eluted from polyacrylamide gels. A total of 85 MS-AFLP polymorphic band pairs of *Hpa*II and *Msp*I (representing 18 loci) were cut from the gels. Of these, 41 pairs (15 loci) were identified as clear bands using agarose gel electrophoresis and were subjected to further cloning. The amplified fragments were ligated to pGEM-T easy vector (Promega, Madison, WI) according to the manufacturer’s instructions, and each clone was sequenced using a BigDye Terminator Cycle Sequencing Ready Reaction Kit (TaKaRa, Shiga, Japan). Finally, from the 207 sequenced samples (12 loci), we identified the sequences of five epigenetic loci. We confirmed that these sequences had the expected lengths and sequences at both ends, and that they were not detected when using the other isoschizomer in the alternate MS-AFLP. The five sequences were annotated based on sequence similarity using the Basic Local Alignment Search Tool of the DNA Data Bank of Japan. All five sequences showed similarity with parts of the reported sequences of close relatives *Arabidopsis* and *Brassica* (Table S3).
