# Supplementary material for: Genet-specific DNA methylation probabilities detected in a spatial epigenetic analysis of a clonal plant population
Source: PLoS One. 2017 May 22;12(5):e0178145. doi: 10.1371/journal.pone.0178145 (PMC5439711; doi:10.1371/journal.pone.0178145)
Supplement: S3 Appendix — (DOCX) [file pone.0178145.s010.docx]

**S3 Appendix. JAGS code.**

#Observation

for (i in 1:N.sample) {

for (type in 1:N.type) {

Methylation[i] ~ dbern(q[i, type])

logit(q[i, type]) <- (

locus[Locus[i], type]

+ cover[Locus[i], type] * Cover[i]

+ geno.locus[Genotype[i], Locus[i], type]

+ block[Locus[i], type, Grid[i]]

)

}

}

#Parameters

Tau.noninformative <- 1.0E-04

Sigma.max <- 100

for (type in 1:N.type) {

mean.locus[type] ~ dnorm(0.0E+00, Tau.noninformative)

mean.cover[type] ~ dnorm(0.0E+00, Tau.noninformative)

for (lc in 1:N.locus) {

locus[lc, type] ~ dnorm(mean.locus[type], tau[1])

cover[lc, type] ~ dnorm(mean.cover[type], tau[2])

}

for (gt in 1:N.genotype) {

for (lc in 1:N.locus) {

geno.locus[gt, lc, type] ~ dnorm(0.0E+00, tau[3])

}

}

for (lc in 1:N.locus) {

for (grid in 1:N.grid) {

block[lc, type, grid] ~ dnorm(0.0E+00, tau[3 +

type])

}

}

}

for (k in 1:N.sigma) {

tau[k] <- 1/(sigma[k] * sigma[k])

sigma[k] ~ dunif(0.0E+00, Sigma.max)

}
